# Choline Transporter regulates olfactory habituation via a neuronal triad of excitatory, inhibitory and mushroom body neurons

**DOI:** 10.1371/journal.pgen.1009938

**Published:** 2021-12-16

**Authors:** Runa Hamid, Hitesh Sonaram Sant, Mrunal Nagaraj Kulkarni

**Affiliations:** Centre for Cellular and Molecular Biology, Council of Scientific and Industrial Research (CSIR-CCMB), Hyderabad, India; Universidad de Valparaiso, CHILE

## Abstract

Choline is an essential component of Acetylcholine (ACh) biosynthesis pathway which requires high-affinity Choline transporter (ChT) for its uptake into the presynaptic terminals of cholinergic neurons. Previously, we had reported a predominant expression of ChT in memory processing and storing region of the *Drosophila* brain called mushroom bodies (MBs). It is unknown how ChT contributes to the functional principles of MB operation. Here, we demonstrate the role of ChT in *Habituation*, a non-associative form of learning. Odour driven habituation traces are laid down in ChT dependent manner in antennal lobes (AL), projection neurons (PNs), and MBs. We observed that reduced habituation due to knock-down of ChT in MBs causes hypersensitivity towards odour, suggesting that ChT also regulates incoming stimulus suppression. Importantly, we show for the first time that ChT is not unique to cholinergic neurons but is also required in inhibitory GABAergic neurons to drive habituation behaviour. Our results support a model in which ChT regulates both habituation and incoming stimuli through multiple circuit loci via an interplay between excitatory and inhibitory neurons. Strikingly, the lack of ChT in MBs shows characteristics similar to the major reported features of Autism spectrum disorders (ASD), including attenuated habituation, sensory hypersensitivity as well as defective GABAergic signalling. Our data establish the role of ChT in habituation and suggest that its dysfunction may contribute to neuropsychiatric disorders like ASD.

## Author summary

Habituation is a conserved phenomenon that enables an organism to enhance attention only on salient stimuli in the surroundings and ignore stimuli without any positive or negative consequences. The circuitry, regulators, and molecular mechanisms involved in habituation are poorly understood. By using *Drosophila* model system, we demonstrate that Choline Transporter (ChT) regulates olfactory habituation and its central operational features. We show for the first time that ChT is localised in GABAergic neurons and demonstrate that reduced levels of ChT in mushroom body and GABAergic neurons lead to defective habituation, which is correlated to augmented sensory perception. Our data provides a new perspective of ChT in habituation and provides avenues for future research to investigate the molecular correlates of ChT in habituation-associated neuropsychiatric disorders.

## Introduction

Acetylcholine (ACh) is the fundamental neurotransmitter of cholinergic neurons. These neurons are widely distributed throughout the central nervous system (CNS) in the vertebrate and invertebrate brain. In vertebrates, all pre-ganglionic sympathetic neurons, part of post-ganglionic sympathetic neurons, pre and post ganglionic parasympathetic neurons are cholinergic [1]. Also, in invertebrates like *Drosophila*, almost all major types of sensory neurons, including chemosensory, chordotonal, olfactory neurons and most regions of the central brain and interneurons are cholinergic [2–4]. Given the widespread distribution of cholinergic neurons in the vertebrate and invertebrate brain, ACh mediated neurotransmission is crucial for neural functions that include varied sensory modalities.

ACh synthesis for efficient neurotransmission at cholinergic synapses depends on the proteins of its metabolic cycle, namely, Choline acetyltransferase (ChAT), vesicular acetylcholine transferase (VAChT), Acetylcholine esterase (AChE) and ChT. The ACh is synthesized by enzyme ChAT from choline and acetylcoenzymeA. It is then transported into synaptic vesicles by VAChT. The ChT imports choline into the presynaptic terminal and is the rate-limiting step of ACh biosynthesis. The MBs in *Drosophila* CNS are bilateral neuropilar areas having evolutionary similarities with vertebrate cortex [5]. *Drosophila* MBs express ChAT and VAChT and require ACh for its function [6,7]. Based on immunostainings, we recently reported a preponderance of ChT in *Drosophila* MBs as compared to ChAT and VAChT [8]. This finding was intriguing which led us to explore why ChT has preferentially higher expression in MBs as compared to ChAT and VAChT. MBs receive olfactory input from the antennal lobes (AL) via projection neurons (PNs) and serve as the prime site for sensory integration and learning. MBs associate the memory with a reward or punishment pathway. However, before establishing such an association in MBs, an animal must evaluate each incoming stimulus, identify the salient stimuli and initiate the appropriate stimulus driven response. Therefore, to understand the mechanisms of complex associative learning, it is important to decipher the mechanisms in MBs that impart an animal the flexibility to choose only the salient incoming stimuli, ignore inconsequential ones and finally register this information in a context-dependent manner. ‘Habituation’ is one such behavioral process that enables an organism to evade inconsequential stimuli from the salient ones. Several reported *Drosophila* genes like *dunce*, *rutabaga*, *turnip*, *radish*, *DCO*, *Leonardo*, *DAMB*, *Nmdar1 and 2* have high expression in MBs and contribute to *Drosophila* memory [9]. Many of the proteins expressed at elevated levels in the MBs are required for both associative learning and habituation [10,11], suggesting that the proteins contributing to associative learning also contribute to habituation. This implies the existence of an association between the two forms of plasticity. Flies that lack MBs display reduced habituation [10,12,13]. Also, habituation to repetitive footshock stimuli requires intact MBs [14]. Thus, these reports signify the importance of MB function in habituation behaviour.

Habituation enhances attention only on the salient features of the animal’s surroundings such as food, mate, danger, etc. Habituation has been observed in many organisms from as simple as a single cell protozoa to *Aplysia californica*, medicinal leech, territorial fish, Birds, and *Drosophila* to more complex life forms like rats, and humans suggesting its ubiquitous persistence [15]. Multiple habituation studies focused on *Drosophila* sensory systems such as olfactory, gustatory, visual, and proprioceptive systems have contributed to our understanding of the cellular and circuit basis of habituation [11,16]. At the synaptic level, habituation may result through the action of heterosynaptic modulation involving activation of inhibitory neurons or homosynaptic depression of excitatory neurons [17]. Studies in adult *Drosophila* suggest that potentiation of GABAergic inhibition onto PN terminals in AL cause olfactory habituation [18]. Habituation reflects the efficient sensory input processing, and defective or premature habituation may lead to sensory hyper-responsivity, which has been widely observed in individuals with Autism Spectrum Disorder (ASD) [19–22]. Recently, orthologs of 98 human intellectual disability (ID) genes were reported to be important for habituation in fruit flies and a large fraction of these genes were associated with ASD [23]. In view of our previous findings that report high expression of ChT in MBs, we study a putative role of ChT in ‘Habituation’ which is widely regarded as a prerequisite for more complex form of associative learning. Compilation of the previous information describing habituation in *Drosophila* reveals that most of the olfactory habituation paradigms engage olfactory circuitry [24,25]. Therefore, we mapped the function of ChT in MB neurons as well as in the olfactory pathway governing olfactory habituation in *Drosophila* larvae.

Here, we show that ChT is required for olfactory habituation at multiple loci involving olfactory pathway and MB neurons. This study reports for the first time that ChT is also localised in GABAergic terminals of *Drosophila* larval brain suggesting that ChT is not unique to cholinergic neurons. We show that the olfactory PN terminals (majority of which are excitatory) and the inhibitory GABAergic neuron terminals express ChT and perhaps their teamwork regulates habituation and its central operational features: the response devaluation to an olfactory stimulus, spontaneous recovery on the removal of stimulus or dishabituation of response upon exposure to an unrelated stimulus. Knock-down of ChT in MBs was observed to be correlated with the hypersensitivity towards the incoming stimuli and defective habituation, suggesting that ChT bridges the link between upstream plasticity and downstream stimulus suppression. Our results demonstrate the role of a conserved protein, ChT, contributing to the dynamic nature of habituation and highlight that its dysfunction leads to sensory abnormalities. Thus, these findings add insight into habituation behaviour mechanisms at the neural circuit levels through choline metabolism.

## Results

### Decrement of odour-specific chemotactic response in naïve *Drosophila* larvae conforms to habituation parameters

Attraction towards an odour is referred to as chemotaxis, which is essential for a diversity of insects to navigate for food sources, potential mating partners, assessing danger, search for egg-laying sites, etc. The olfactory system of insects has evolved to impart them great discriminatory power for behaviourally relevant odours, decipher this message in CNS and finally exhibit appropriate behaviours. Thus, chemotaxis-based behavioural responses towards an odour are significant for their survival. Foraging *Drosophila* larvae are in constant search of food for their development. The larval olfactory system is similar but numerically simpler than the adult flies, discern a wide range of odours and learn to discriminate between different odours and concentrations [26–29]. Thus, it provides a system with a genetically accessible, well-defined neural circuit relevant to our study.

**Fig 1:**
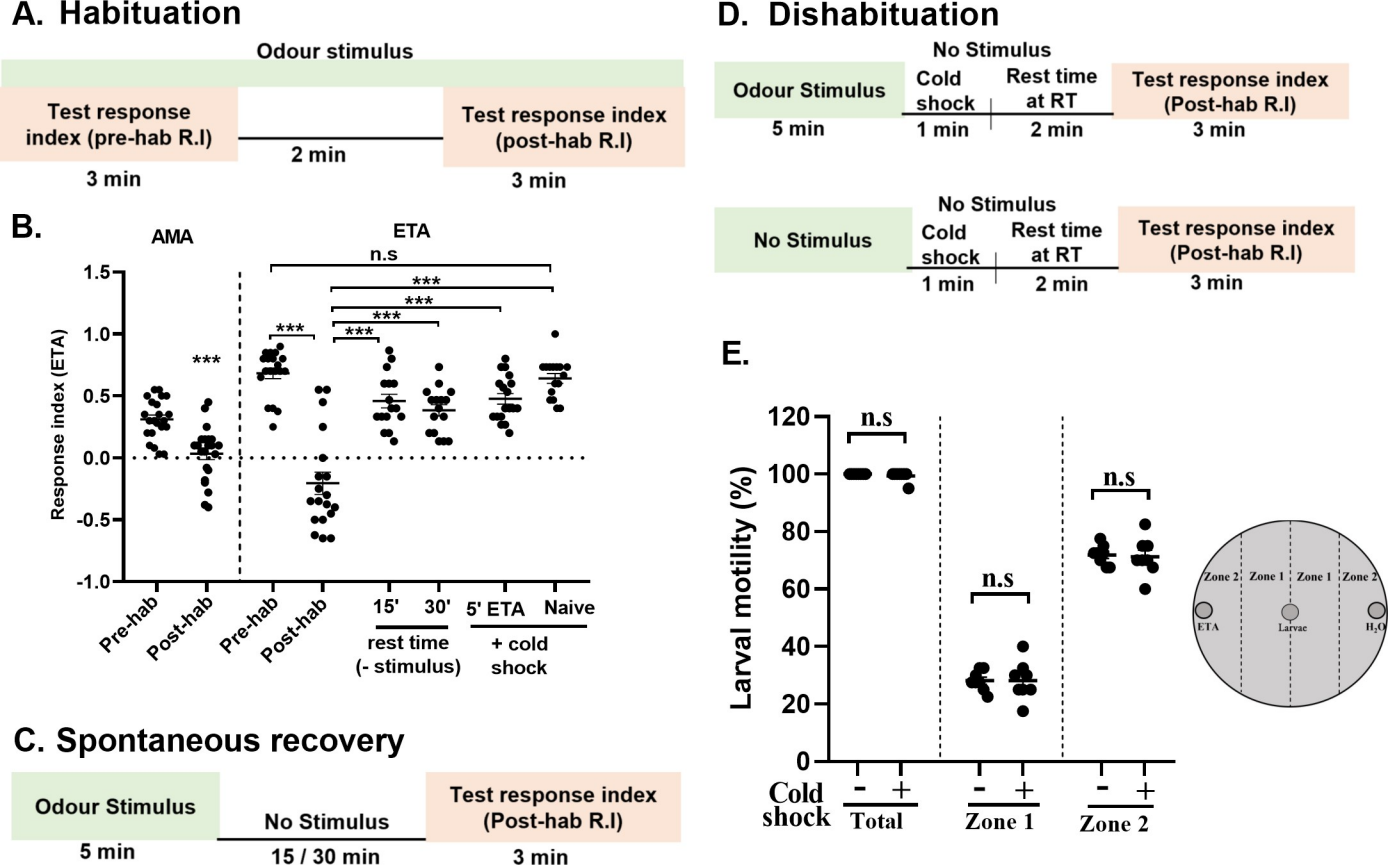
Olfactory habituation assay in wild-type *Drosophila* larvae. (A) Schematics represent the specific time segments to induce olfactory habituation in wild-type (W^1118^) larvae. Naïve larvae were exposed to the odour and response index (R.I) was calculated after 3 min (Pre-hab R.I). The same set of larvae were left for additional 2 min in presence of odour for habituation (This makes total odour exposure time to 5 min). The odour exposed larvae were brought back in the middle of the petriplate and the R.I was again calculated (Post-hab R.I). (B) Scatter plot shows response indices (R.I, black circles) of naïve wild type larvae towards amyl acetate (AMA) or ethyl acetate (ETA) (pre-hab R.I) and after 5 min of odour exposure (post-hab R.I). Larvae pre-exposed to odour for 5 min shows spontaneous recovery after 15 or 30 min in the absence of stimulus (*see spontaneous recovery schematics*). Larvae pre-exposed to odour for 5 min shows recovery by 1min cold shock (5’ETA+cold shock), the phenomenon called dishabituation (*see dishabituation schematics)*. R.I of naïve larvae without odour pre-exposure but exposed to cold shock (Naïve+cold shock) is not altered due to cold shock. (C) Schematics represent the specific time segments to induce spontaneous recovery, (D) Schematics represent the specific time segments to induce dishabituation. (E) The scatter plot shows percent larval motility of 1 min cold shock treated (+) and untreated larvae (-), in a petriplate with ETA and water on opposite ends. As shown in the schematics on right, the larvae were kept in the centre and after 3 min, the total number of larvae that moved to different zones were counted. The data represented here shows percentage of total larvae moved, larvae moved to zone1, and zone 2, N = 8. Data is represented as scatter plot with error bars showing SEM and N≥16, unless mentioned. Each N represent one experiment performed with a group of 30–40 larvae. Each data group was analysed for normal distribution using Shapiro-Wilk test. Statistical significance was determined by two-tailed unpaired t-test (parametric) with Welchs correction. *** represent p≤0.0001, n.s means statistical non-significance when p≥0.05. For statistical details and numerical data values in the scatter plot refer to S1 and S2 Data.

We first tested and standardised the habituation assay in wildtype 3^rd^ instar foraging larvae to affirm if our assay accedes to habituation parameters. Naïve wildtype larvae were attracted by Ethyl acetate (ETA) and Amyl acetate (AMA) as assessed by calculating response index (Pre-hab R.I) (Fig 1A and 1B). Continuous exposure of naïve wild type larvae for 5 min to AMA and ETA, evoked significant avoidance of these odours (Post-hab R.I) (Fig 1A and 1B). However, among both the odours tested, ETA elicited a more robust chemotactic response and showed stronger avoidance after prior exposure. Therefore, we used the attractant ETA for subsequent experiments. We further investigated whether the decrement of chemotactic response conforms to classical habituation parameters [30], which means animals that habituate to a stimulus should regain the response after a time-lapse, if the stimulus is withheld. This phenomenon is termed as ‘spontaneous recovery’ (Fig 1C). Indeed, we observed a spontaneous recovery of the chemotactic response to the naïve levels after 15 and 30 min rest time (Fig 1B). To confirm the initial decrement as habituation and not a fatigue or sensory adaptation, we tested another classical habituation feature called ‘Dishabituation’. It means if the habituated animal is exposed to an unrelated strong stimulus, the naïve response is fully or partially restored. We attempted dishabituation using a 1 min exposure to cold shock on ice (*Schematics*, Fig 1D). This significantly reverses the chemotactic response of larvae pre-exposed to 5’ ETA (Fig 1B). Notably, 1 min exposure to cold shock given to naïve larvae did not affect R.I towards ETA as compared to pre-hab R.I, suggesting that cold shock does not affect the general perception of olfactory stimuli or cause sensitisation (Fig 1B). We also assessed effect of 1 min cold shock on larval motility before and after the cold shock and observed that 100 percent larvae had left the choice point (Fig 1E). Under both conditions (with cold shock and without cold shock), 28 percent larvae moved to zone 1 and 71 percent larvae moved to zone 2 (Fig 1E), suggesting that 1 min cold shock does not have any effect on larval motility.

Taken together, the decrement of chemotaxis in wildtype larvae on continuous exposure to an odorant stimulus, the recovery of the response when the stimulus was withheld, and the dishabituation conforms to the habituation parameters and demonstrates that the response attenuation is attributed to olfactory habituation. We have used these olfaction-based paradigms to demonstrate the functional relevance of ChT in regulating habituation in our subsequent experiments.

### Intrinsic neurons of MB require ChT for olfactory habituation and incoming odour stimulus suppression

**Fig 2:**
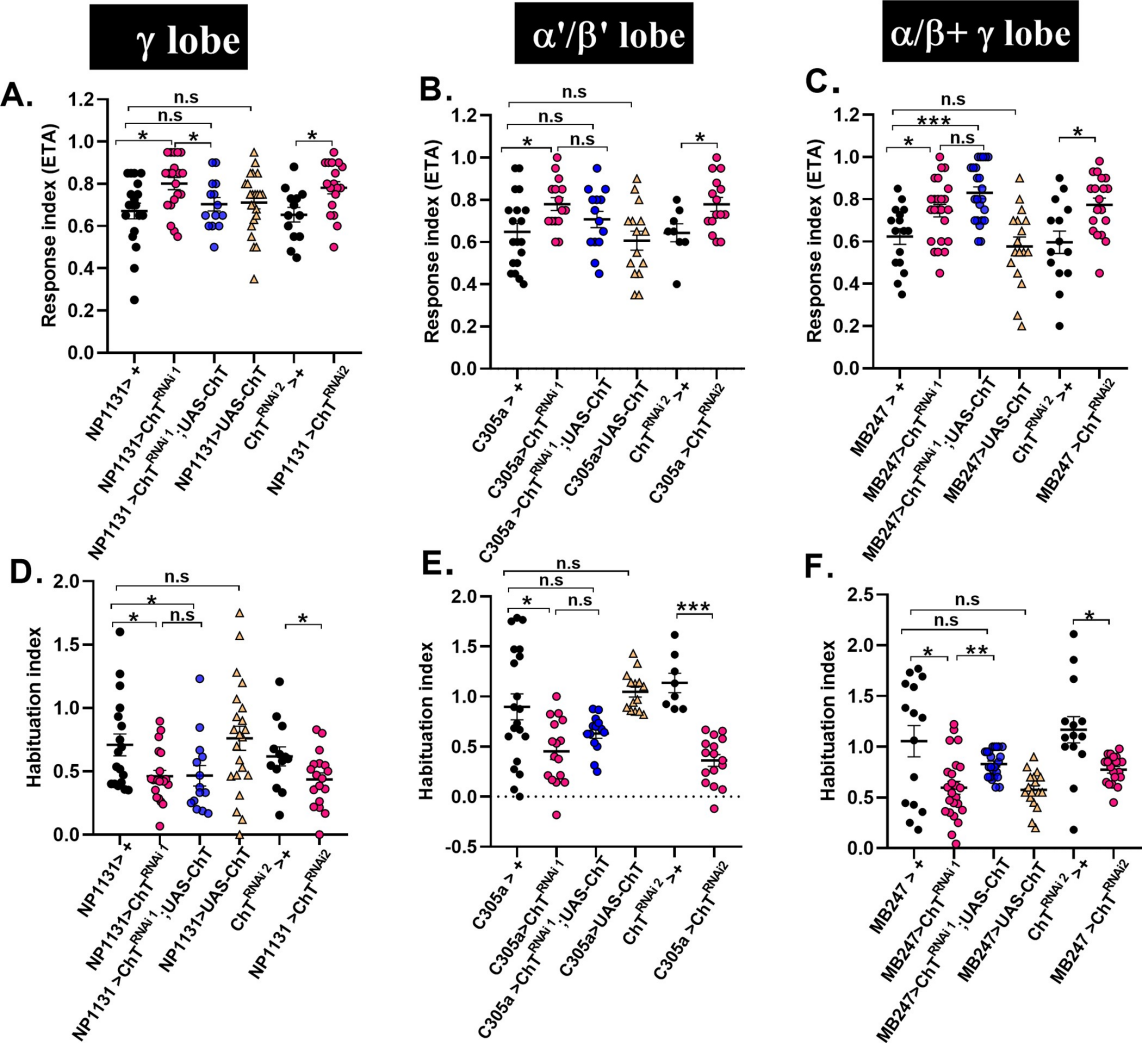
Knock-down of ChT in MB intrinsic neurons enhances chemotaxis towards odour but suppresses habituation. (A-C) Response index of naïve larvae (*also referred to as chemotaxis*) towards ETA, (D-E) habituation index of larvae exposed to ETA, of genotypes: NP1131GAL4 was used as a driver line for expression in MB **γ**-lobe. Scatter plot represents R.I (A) and H.I (D) of genotypes *NP1131*> *UAS-ChT*^*RNAi1*^, *NP1131>UAS-ChTRNAi1;UAS-ChT*, *NP1131*> *UAS-ChT* as compared to their controls *NP1131*> *+*. *NP1131*> *UAS-ChT*^*RNAi2*^ as compared to *UAS-ChT*^*RNAi2*^*>+*. C305aGAL4 was used as a driver line for expression in MB α’/β’-lobe. Scatter plot represents R.I (B) and H.I (E) of genotypes *C305a*> *UAS-ChT*^*RNAi1*^, *C305a> UAS-ChTRNAi1;UAS-ChT*, *C305a*> *UAS-ChT* as compared to their controls *C305a*> *+*. *C305a*> *UAS-ChT*^*RNAi2*^ as compared to *ChT*^*RNAi2*^*>+*. MB247GAL4 was used as a driver line for expression in MB α/β+ **γ** lobe. Scatter plot represents R.I (C) and H.I (F) of genotypes *MB247*> *UAS-ChT*^*RNAi1*^, *MB247> UAS-ChTRNAi1;UAS-ChT*, *MB247*> *UAS-ChT* as compared to their controls *MB247*> *+*. *MB247*> *UAS-ChT*^*RNAi2*^ as compared to *UAS-ChT*^*RNAi2*^*>+*. Pink circles represent knockdown using *UAS-ChT*^*RNAi*^*1* and *UAS-ChT*^*RNAi2*^, Blue circles represent rescue, and yellow triangles represent transgenic over-expression of *UAS-ChT* as compared to genetic controls (black circles). Data are represented as scatter plot with error bars showing SEM and N≥16. Each N represent one experiment performed with a group of 40 larvae. Each data group was analysed for normal distribution using Shapiro-Wilk test. Statistical significance was determined by two-tailed unpaired t-test (parametric) with Welchs correction. *** represent p≤0.0001, n.s means statistical non-significance when p≥0.05. For more statistical details and numerical data values in the scatter plot refer to S1 and S2 Data.

There are three types of MB intrinsic neurons, i.e., α/β, α’/β’ and **γ** neurons, also termed as Kenyon cells (KC). These are distinctly implicated in olfactory learning and memory [31,32]. To understand the role of ChT in MBs, we depleted it in independent domains of MB intrinsic neurons with the help of *UAS-GAL4* binary expression system [33]and investigated if these neurons require ChT function in habituation. Our immunostainings show ChT colocalization in MB calyx and different domains of MB neurons as assessed by driving expression of UAS-mCD8GFP with MB247 (α/β + **γ** class of KC) and C739 (α/β class of KC), C305a (α’/β’ class of KC), and NP1131 (**γ** class of KC) (S2 Fig). We used two RNAi fly stocks [*ChT*^*RNAi1*^ (V101485) and *ChT*^*RNAi2*^ (BL28613)] to knock down ChT in MB intrinsic neurons. The knock-down efficiency of *ChT*^*RNAi2*^ was assessed by immunostaining (S1 Fig), while that of ChT^RNAi1^ was described previously by us [8]. First, we evaluated the naïve chemotactic response of 3^rd^ instar foraging larvae towards ETA upon knock-down of *ChT* in each of the neuronal domains of MBs. Interestingly, we observed a significant increase in naïve chemotactic response upon knock-down of ChT in **γ** lobe neurons (NP1131> *ChT*^*RNAi1*^ *or ChT*^*RNAi2*^) as well as in α’/β’ (C305a> *ChT*^*RNAi1*^ *or ChT*^*RNAi2*^) and α/β + **γ** (MB247> *ChT*^*RNAi1*^ *or ChT*^*RNAi2*^) class of KC as compared to their genetic controls (Fig 2A–2C). To test if chemotactic enhancement is specific to the knock-down of ChT, we expressed ChT transgene on *UAS-ChT*^*RNAi1*^ background in all the three neuronal domains of MBs (*MBGAL4s> ChT*^*RNAi1*^*;UAS-ChT*) and observed a reversal of the response index in **γ** and α’/β’ class of KC but not in α/β+ **γ** class of KC when compared with respective genotypes of MBGAL4s> + (Fig 2A–2C). Additionally, we compared response index of genotype *MBGAL4s> ChT*^*RNAi1*^*;UAS-ChT* with *MBGAL4s> ChT*^*RNAi1*^ but observed statistically significant reversal of the response index only in **γ** lobe but not in α’/β’ and α/β+ **γ** class of KC (Fig 2A–2C). Next, we tested the effect of ChT knock-down in MBs on olfactory habituation. A significant reduction in habituation index was observed in the group of larvae upon knock-down of ChT with both *ChT*^*RNAi1*^ *and ChT*^*RNAi2*^ fly lines in **γ**, α’/β’ and α/β+ **γ** class of KC as compared to their genetic controls (Fig 2D–2F). Transgenic expression of ChT in *UAS-ChT*^*RNAi1*^ background significantly enhanced the habituation index of the larvae in α’/β’ and α/β+ **γ** class of KC but not in **γ** KC (Fig 2D–2F). This difference in the rescue of response index and habituation index in different KC may be due to the differences in the expression levels of *UAS-ChT*^*RNAi1*^ and *UAS-ChT* transgenes driven by the different GAL4s. To clarify, if the levels of ChT determine the extent of habituation and chemotaxis, we over-expressed ChT in all the three classes of KC neurons using specific GAL4 drivers (MBGAL4’s>*UAS-ChT*). The extent of the naïve olfactory response, as well as the habituation, remains unaffected by overexpression of ChT (Fig 2A–2F). Next, we confirmed if larvae’s enhanced response towards ETA and decreased habituation is specific to the knock-down of ChT and not specific to the kind of pre-exposed odour. For this, we knocked down ChT in **γ**, α’/β’ and α/β+ **γ** KC using *UAS-ChT*^*RNAi*1^ (*MBGAl4s>ChT*^*RNAi1*^) and exposed these group of larvae to amyl acetate (AMA) and the alcoholic class of odour, 3-Octanol. We observed an enhancement of chemotaxis and reduced habituation for both the pre-exposed odours, suggesting it to be ChT specific phenotype and not to the class of odour (S3A–S3D Fig).

To investigate if the synaptic transmission from MBs is required to regulate incoming odour stimulus and habituation, we expressed the temperature-sensitive mutant of Dynamin orthologue, *Shibire*, in MB neurons using a *UAS-Shi*^*ts*^ transgene. *Shibire*^*ts*^ mutant causes a block of synaptic vesicle recycling at non-permissible temperature (29°C), leading to a rapid decline of neurotransmitter release and synaptic transmission [34]. The neural activity was perturbed specifically in α’β’, γ and αβ+ **γ** class of KC intrinsic neurons using *C305a*-GAL4, *NP1131*-GAL4 and *MB247*-GAL4, respectively, and their consequent effect on the naïve chemotactic response and habituating ability of the larvae at non-permissible temperature was assessed. A significantly enhanced chemotaxis towards ETA and reduced habituation were observed as compared to their genetics controls (GAL4s>+) (S4A and S4B Fig). Inactivation of synaptic vesicle endocytosis in MB intrinsic neurons resulted in enhanced chemotaxis and attenuated habituation similar to those observed with knock-down of ChT in these neurons. This suggests a need for neurotransmitter release from MBs for incoming odour stimulus suppression and habituation.

Previously, we reported attenuated neuromuscular junctions (NMJ) in third instar larvae due to knock-down of ChT in α/β and **γ** intrinsic lobes of MB [8]. To ascertain if the observed reduction in habituation index is the acute functional change in the neurons during habituation or has resulted from changes in the NMJ, we employed the TARGET system [35]. TARGET system uses a Tubulin promotor to express temperature-sensitive repressor of GAL4 (TubGAL80^ts^) where expression of GAL80^ts^ allows RNAi expression by GAL4 at 29°C but not at 18°C. Using this system, we limited the expression of *UAS-ChT*^*RNAi1*^(*MBGAL4>UAS-ChT*^*RNAi1*^*;tubGAL80*^*ts*^) to the developmental window of foraging 3^rd^ instar larvae during which the assay was performed (S5A Fig). We observed a significant enhancement in response index towards the odour (S5B Fig) and a drastic reduction in habituation index (S5C Fig) upon knock-down of ChT in α/β and **γ** lobe neurons at 29°C but not at 18°C (S5B and S5C Fig). Reportedly, *MB247GAL4* has expression domain in both **γ** and α/β domain [36]. Therefore, we validated our results obtained with *MB247GAL4* by performing experiments with additional GAL4 line, *C739GAL4*, which preferentially expresses only in α/β domain [36]. We observed an enhanced response index and reduced habituation index as compared to their genetic controls at 29°C but not at 18°C (S5D and S5E Fig). These results confirm that ChT is acutely required in MB intrinsic neurons to facilitate habituation and the observed habituation defects are not due to changes in neurons during development. Altogether, our results indicate that knock-down of ChT in MB does not affect the odour perception. It has a function in MB intrinsic neurons that support devaluation of the incoming stimuli and regulates sensitivity towards incoming olfactory stimuli.

### ChT is essential in intrinsic neurons of MB for the maintenance of key characteristics of habituation

**Fig 3:**
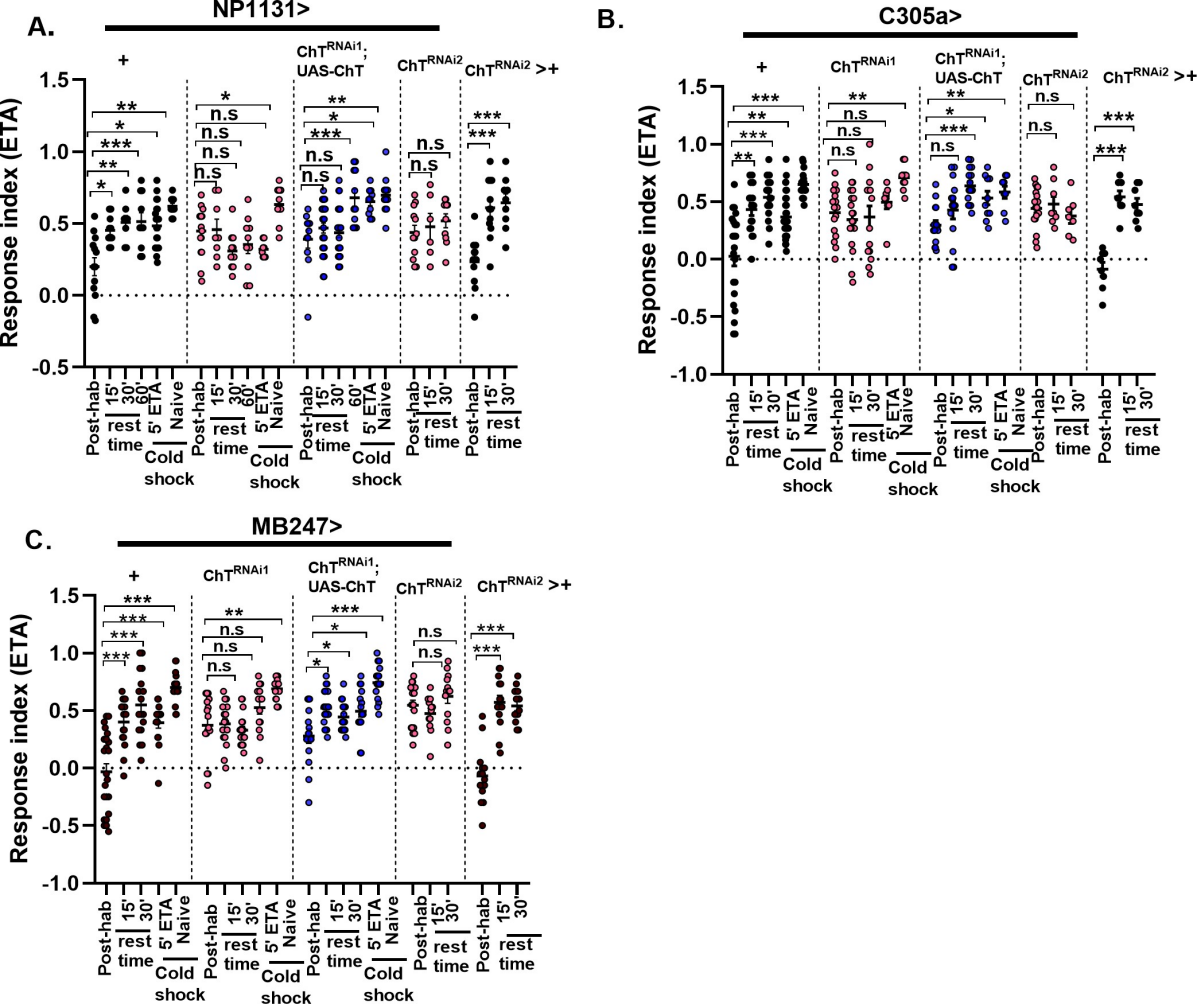
Knock-down of ChT in MB intrinsic neurons impairs spontaneous recovery and dishabituation of habituated larvae. Scatter plot shows response indices towards ETA of larvae pre-exposed to 5 min ETA (Post-hab). For spontaneous recovery, the larvae were pre-exposed to 5’ETA and R.I was calculated after 15, 30 or 60 min rest time in absence of stimulus. For dishabituation, the larvae were pre-exposed to 5’ETA and R.I was calculated after 1 min cold shock (5’ETA+coldshock). Scatter plot also shows R.I of naïve larvae presented to 1 min cold shock (naïve+ cold shock). Scatter plot shows R.I in the described conditions in genotypes: (A) *NP1131*> *UAS-ChT*^*RNAi1*^ (pink circles), *NP1131> UAS-ChTRNAi1;UAS-ChT* (blue circles) as compared to their controls *NP1131*> *+* (black circles). *NP1131*> *UAS-ChT*^*RNAi2*^ (pink circles) as compared to *UAS-ChT*^*RNAi2*^*>+* (black circles). (B) *C305a*> *UAS-ChT*^*RNAi1*^ (pink circles), *C305a> UAS-ChTRNAi1;UAS-ChT* (blue circles) as compared to their controls *C305a*> *+*(black circles). *C305a*> *ChT*^*RNAi2*^ (pink circles) as compared to *UAS-ChT*^*RNAi2*^*>+* (black circles). (C) *MB247*> *UAS-ChT*^*RNAi1*^ (pink circles), *MB247> UAS-ChT*^*RNAi1*^*;UAS-ChT* (blue circles) as compared to their controls *MB247*> *+* (black circles). *MB247*> *UAS-ChT*^*RNAi2*^ (pink circles) as compared to *UAS-ChT*^*RNAi2*^*>+* (black circles). Both spontaneous recovery and dishabituation was impaired in ChT knocked-down group of larvae (data represented by pink circles). Each N represent one experiment performed with a group of 30 larvae. Each data group was analysed for normal distribution using Shapiro-Wilk test. Statistical significance was determined by two-tailed unpaired t-test (parametric) with Welchs correction. *** represent p≤0.0001, ** represent p≤0.001, n.s means statistical non-significance when p≥0.05. For more statistical details and numerical data values in the scatter plot refer to S1 and S2 Data.

A habituated animal should re-establish the response towards stimuli partially or wholly within a specific time frame if the stimulus is withheld [30]. To ascertain that the decrement in response due to knock down in ChT is indeed habituation phenotype, we assessed the response recovery (spontaneous recovery), after a rest period of 15 min and 30 min, in the habituated larvae when the stimulus was withheld. The knock-down of ChT using the expression of *UAS-ChT*^*RNAi1*^ and *UAS-ChT*^*RNAi2*^ lines driven by *NP1131GAL4* (**γ** lobe, Fig 3A), *C305aGAL4* (α’/β’lobe, Fig 3B) and *MB247GAL4* (α/β+ **γ** lobe, Fig 3C) intrinsic lobes caused a defect in the larvae to spontaneously recover. On the other hand, larvae from their genetic controls (*MBGAL4s*>+ or *UAS-ChT*^*RNAi2*^*>+*) significantly recovered to the naïve levels after just 15 min (Fig 3A–3C). The defect in recovery due to depletion of ChT was restored partially or completely when *ChT* was transgenically expressed on a *ChT*^*RNAi1*^ background in α’/β’, α/β+ **γ** and **γ** class of KC neurons (Fig 3A–3C). However, we noticed a significant rescue in spontaneous recovery in **γ** lobe only after 60 min whereas in α’/β’ and α/β+ **γ**, the recovery was achieved within 30 min.

Next, we induced dishabituation by exposing the habituated larvae to cold shock for 1 min. The knock-down of ChT in the **γ**, α’/β’ and α/β+ **γ** class of KC neurons of larvae using *NP1131GAL4* (Fig 3A), *C305aGAL4* (Fig 3B) and *MB247GAL4* (Fig 3C) driver lines, respectively, were unable to dishabituate upon cold shock. On the other hand, exposure of habituated larvae to 1 min cold shock reverses chemotaxis decrement to naïve levels in the control group of larvae (*MBsGAL4*>+, Fig 3A–3C). The dishabituating capability was restored in these larvae when ChT was transgenically expressed on a *ChT*^*RNAi*^ background (*UAS-ChT*^*RNAi1*^*;UAS-ChT*) in γ, α’/β’, and α/β class of KC neurons (Fig 3A–3C). The exposure of naïve larvae to similar cold shock did not affect the chemotaxis both in control and ChT-depleted group of larvae, suggesting that the observed enhancement of response index is not the sensitisation of olfactory receptors due to cold shock.

The control group of larvae (*MBsGAL4*>+) are capable of re-establishing baseline response levels that follow an unrelated dishabituating stimulus as well as after a prolonged lapse of time in the absence of stimulus while larvae deficit of ChT function in MB neurons are incapable of resuming the initial response levels. This suggests that in the absence of ChT, the synaptic capability to re-establish the response is compromised, leading to defective spontaneous recovery and dishabituation.

### ChT is localized in the neural circuit of the larval olfactory pathway

**Fig 4:**
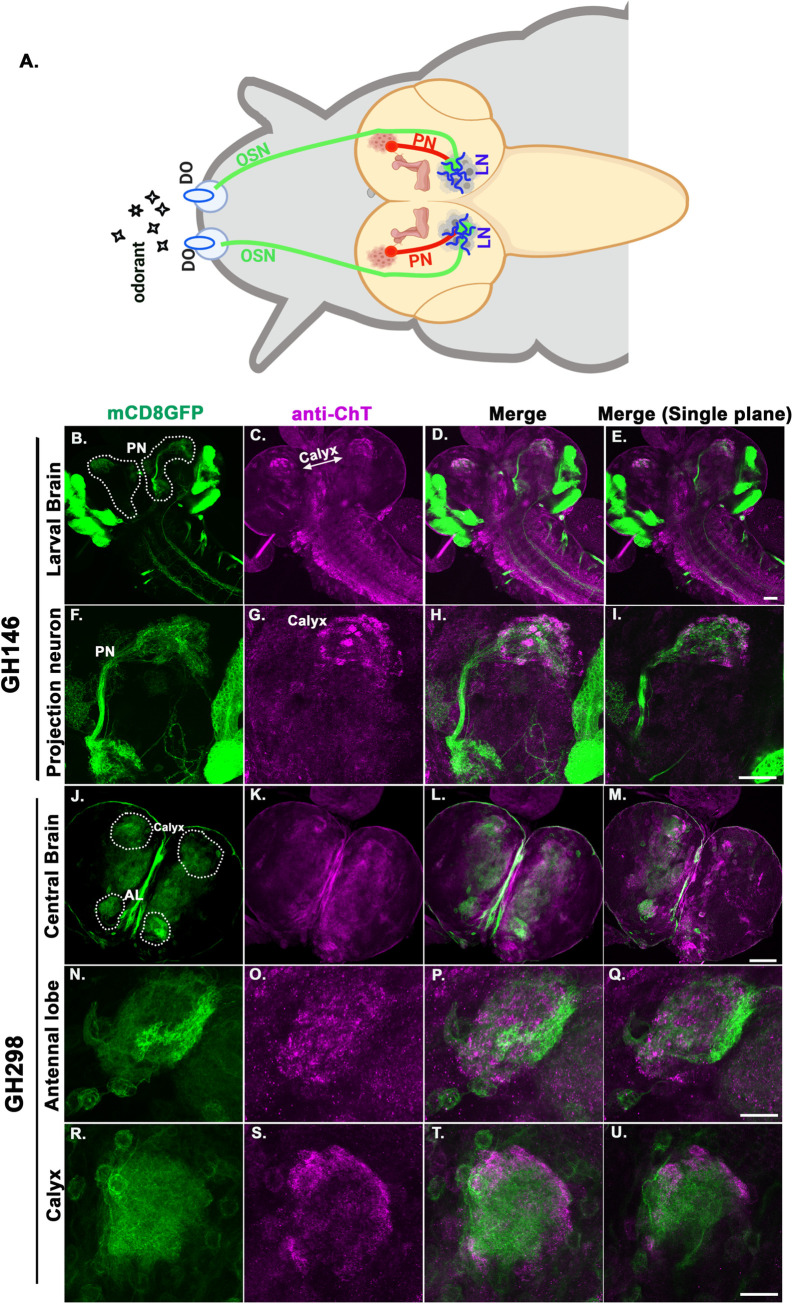
ChT is localised in major neurons of larval olfactory neural circuit. (A) Schematics of the larval olfactory pathway. Odorants are detected by olfactory sensory neurons (OSNs, green), which terminate in antennal lobe (AL) and synapses with local interneurons (LNs, blue) or with dendrites of projection neurons (PNs, red). PNs innervate MB calyx (brown). (B-D) Expression of *UAS-mCD8GFP* in projection neurons (green) driven by *GH146GAL4* and immunostained with anti-ChT (magenta) and merge. The PNs connecting AL to MB calyx are encircled by white dotted shape, scale bar 50 μm. imaged with 20x, 0.9 N.A objective (F-H) PN imaged at higher magnification, scale bar 20 μm, imaged with 40x, 1.4N.A oil objective (E and I) represents the merged image of single plane taken from the same z-section of represented image stacks. The colocalised regions can be viewed as white areas in the merged image shown. (J-M) Expression of *UAS-mCD8GFP* in central brain region (green) driven by *GH298GAL4* and immunostained with anti-ChT (magenta) and merge, scale bar 50 μm. imaged with 20x, 0.9 N.A objective. GH298 shows expression in both AL and calyx. (N-P) AL imaged at higher magnification, scale bar 20 μm, imaged with 40x, 1.4N.A oil objective (R-T) MB calyx imaged at higher magnification, scale bar 20 μm, imaged with 40x, 1.4N.A oil objective. (M, Q, and U) represents the merged image of single plane taken from the same z-section of represented image stacks. The colocalised regions in AL and calyx can be viewed as white areas in the merged image shown. All images are z-stacked (unless mentioned) pseudocoloured representative of 3–5 3^rd^ instar larval brains.

*Drosophila* larva has a well-defined olfactory circuitry where olfactory signals are conveyed through a dome of perforated cuticle called (DO) at its anterior end of the body, which houses 21 olfactory sensory neurons (OSNs). The axons of OSNs bundle together and project into the AL via the antennal nerve [37]. Each OSN expresses a specific type of olfactory receptor (OR) along with the OR83b gene, which is essential for the proper functioning of ORs [38]. The olfactory signals are further conveyed to higher order structures in the brain via PNs [39] (*Schematics* Fig 4A). Previous studies suggest that the potentiation of inhibitory signals from the local interneurons (LNs) present in AL modulate PNs to facilitate olfactory habituation [24]. This led us to hypothesize that ChT might also be present in the olfactory neural circuit and possibly facilitate olfactory habituation. Therefore, we investigated the presence of ChT in OSNs, AL, and PNs using specific GAL4s to drive *UAS-mCD8GFP*. The OSN terminals were visualised by expressing *UAS-mCD8GFP* using *Or83bGAL4* in F1 larval brains of genotype *Or83bGAL4>UAS-mCD8GFP* and costained by the *anti-*ChT antibody. We observed ChT expression only at a few of the termini of its branches and the expression was not as profusely defined as the total number of branches suggesting that its function lies only in a particular subset of OSNs (S6A–S6C Fig). OSNs project their terminals onto the local interneurons in AL or dendrites of projection neurons in AL (*schematics* Fig 4A). PNs are uniglomerular and relay information to MBs or other higher brain regions [40]. They connect to individual glomeruli of calyx via the presynaptic ends and the AL via postsynaptic ends [41]. We used *GH146GAL4* to drive the expression of *UASmCD8GFP* reporter in projection neurons (Fig 4B–4E). The dendritic arborization of PNs covered a larger area of the AL (Fig 4F). An axon extending from AL was visible and terminated in the MB calyx, representing the output area of PNs (Fig 4F). We observed an intense localisation of ChT at the presynaptic ends of PNs terminating in the calyx of MB (Fig 4F–4I). The dendritic end showed a sparse localisation of ChT, which probably represents the expression of ChT in the antennal lobes rather than the dendritic end of PNs (Fig 4G). To identify ChT localisation in the larval antennal lobe neurons, we crossed *GH298GAL4* driver with *UAS-mCD8GFP* and coimmunostained with anti-ChT to visualize ChT expression pattern in AL neurons (Fig 4J–4Q). The *GH298*GAL4 expression was seen as arborisation in AL and in cells situated around the lateral edge of the AL (Fig 4N). GH298 expression was also seen in MB calyx with a predominant colocalization with anti-ChT immunostainings (Fig 4J–4M and Fig 4R–4U). Collectively, our immunostaining data shows that ChT is present in all three major cellular components of the olfactory pathway i.e the OSNs, the AL, the PNs as well as in the MBs of *Drosophila* larva. Therefore, it became logical to investigate the role of ChT in the whole olfactory neural circuitry and assess if ChT contributes at each stage of olfactory processing to facilitate olfactory habituation.

### *ChT* mediates habituation but not chemotaxis through local interneurons of antennal lobes and projection neurons

Our immunostaining analysis shows ChT localisation in olfactory circuit neurons including OSNs, local neurons of AL, PNs and MB intrinsic neurons, which provided the basis to dissect the potential contribution of ChT in each of these kinds of neurons in the olfactory neural circuitry. We first knocked down ChT in OSNs by expressing *UAS-ChT*^*RNAi*1^ using *Or83b*GAL4 (*OR83b*GAL4>*UAS-ChT*^*RNAi*^), acutely knock down ChT in 3^rd^ instar developmental window using TubGAL80^ts^, and knockdown ChT throughout development using TubGAL80^ts^. We observed a reduction in chemotaxis towards ETA in genotype *OR83b*GAL4>*UAS-ChT*^*RNAi*^ and *OR83b*GAL4> *UAS-ChT*^*RNAi*^; *TubGAL80ts* (throughout 29°C) as compared to the genetic control (*OR83b*GAL4>+) (S6D Fig) and not when it was knocked down in the 3^rd^ instar developmental window. This suggests that the reduction observed in chemotaxis arises due to developmental defect in these neurons. The attenuated chemotaxis was partially rescued by transgenically expressing *UAS-ChT* on a *ChT*^*RNAi*^ background (*OR83bGAL4>UAS-ChT*^*RNAi*^*; UAS-ChT*) (S6D Fig). However, we did not find any significant reduction in habituation index neither upon acute knock-down of ChT nor throughout knockdown of ChT (S6E Fig). The overexpression of ChT in OSNs neither modulated chemotaxis nor the larvae’s habituating capability (S6D and S6E Fig). These results imply that ChT localised in OSNs does not have a role in habituation.

**Fig 5:**
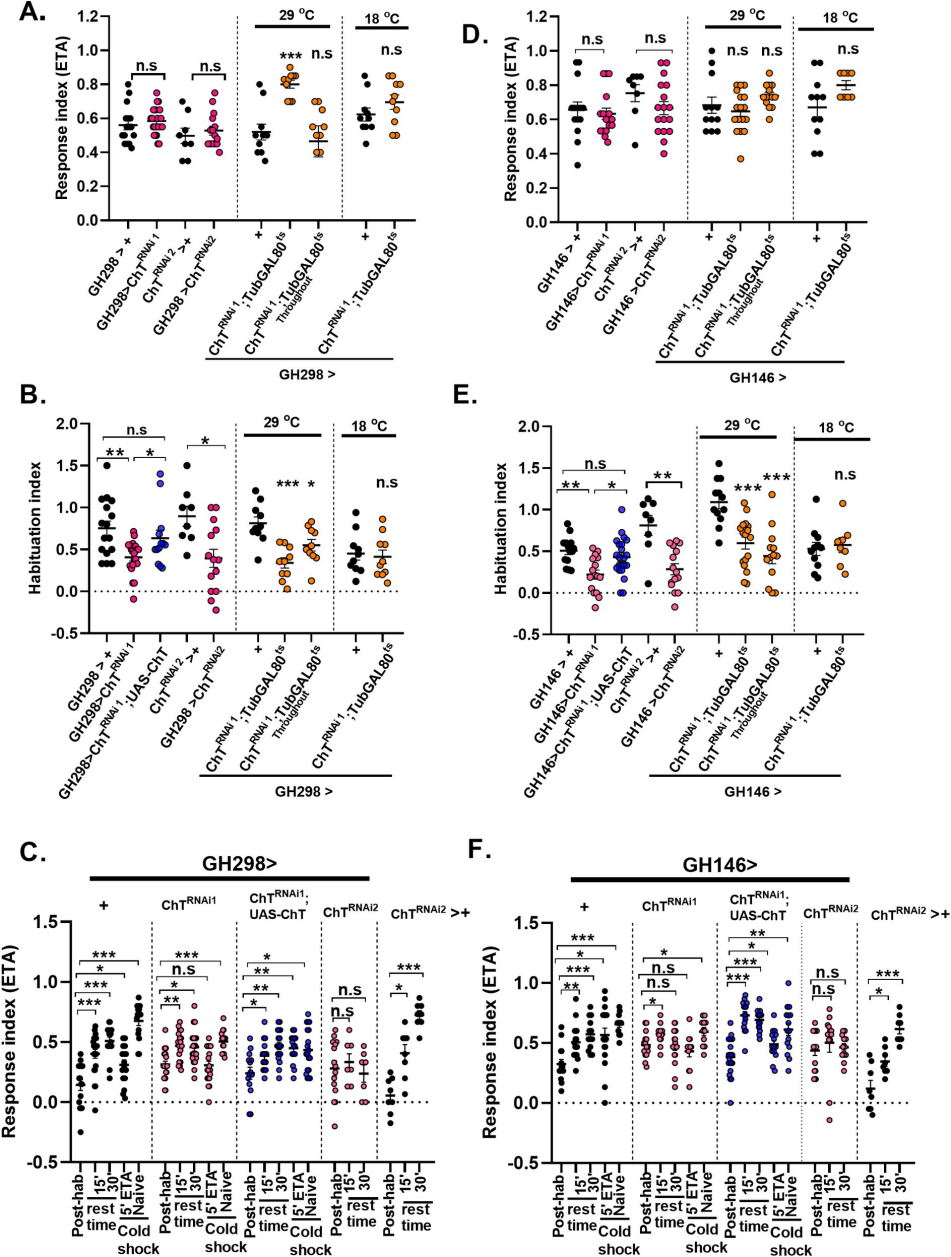
Presence of ChT in antennal lobes and projection neurons is required for olfactory habituation. *GH298GAL4* was used as a driver line for expression in AL (A) response index, (B) habituation index of genotypes: *GH298*> *UAS-ChT*^*RNAi1*^ (pink circles), *GH298> UAS-ChT*^*RNAi1*^*;UAS-ChT* (blue circles) as compared to its control *GH298*> *+* (black circles). *GH298*> *UAS-ChT*^*RNAi2*^ (pink circles) as compared to *UAS-ChT*^*RNAi2*^*>+* (black circles). *GH298>ChT*^*RNAi1*^*;TubGAL80ts* at 29°C where ChT was knocked down only in 3^rd^ instar foraging developmental window or throughout 29°C (orange circles) as compared to *GH298*>+ (black circles). Scatter plot also shows data of genotypes *GH298>ChT*^*RNAi1*^*;TubGAL80*^*ts*^ at 18°C (orange circles) as compared to *GH298*>+ (black circles) at 18°C. Chemotaxis remains unaltered except in the condition when ChT was knocked down only in 3^rd^ instar developmental window while a significant reduction in habituation index was observed when ChT was knocked down in AL neurons marked by *GH298* driver line. (C) response index following 5’ ETA exposure (post-hab), spontaneous recovery in the ETA pre-exposed larvae after 15 and 30 min rest time in the absence of stimulus, dishabituation in the ETA pre-exposed larvae presented to 1 min cold shock (5’ETA+coldshock) and naïve larvae presented to 1 min cold shock (naïve + cold shock), of the genotypes: *GH298*> *+* (black circles), *GH298*> *UAS-ChT*^*RNAi1*^ (pink circles), *GH298> UAS-ChT*^*RNAi1*^*;UAS-ChT* (blue circles), *GH298*> *UAS-ChT*^*RNAi2*^ (pink circles) and *UAS-ChT*^*RNAi2*^*>+* (black circles). Knock-down of ChT impairs spontaneous recovery and dishabituation when ChT was knock down with *GH298GAL4* driver. *GH146GAL4* was used as a driver line for expression in PN. (D and E) shows response index and habituation index, respectively, in genotypes: *GH146*> *UAS-ChT*^*RNAi1*^ (pink circles), *GH146> UAS-ChT*^*RNAi1*^*;UAS-ChT* (blue circles) as compared to its control *GH146*> *+* (black circles). *GH146*> *UAS-ChT*^*RNAi2*^ (pink circles) as compared to *UAS-ChT*^*RNAi2*^*>+* (black circles).*GH146>ChT*^*RNAi1*^*;TubGAL80*^*ts*^ at 29°C where ChT was knocked down only in 3^rd^ instar foraging developmental window or throughout 29°C (orange circles) as compared to *GH146*>+ (black circles). Scatter plot also shows data of genotypes *GH146>ChT*^*RNAi1*^*;TubGAL80ts* 18°C (orange circles) as compared to *GH146*>+ (black circles) at 18°C. A significant reduction in habituation index was observed while chemotaxis remains unaltered when ChT was knocked down in PNs. (F) response index following 5’ ETA exposure (post-hab), spontaneous recovery in the ETA pre-exposed larvae after 15 and 30 min rest time in the absence of stimulus, dishabituation in the ETA pre-exposed larvae presented to 1 min cold shock (5’ETA+coldshock) and naïve larvae presented to 1 min cold shock (naïve + cold shock), of the genotypes: *GH146*> *+* (black circles), *GH146*> *UAS-ChT*^*RNAi1*^ (pink circles), *GH146> UAS-ChT*^*RNAi1*^*;UAS-ChT* (blue circles), *GH146*> *UAS-ChT*^*RNAi2*^ (pink circles) and *UAS-ChT*^*RNAi2*^*>+* (black circles). Knock-down of ChT impairs spontaneous recovery and dishabituation when ChT was knock down in PNs with *GH146GAL4* driver. Each N represent one experiment performed with a group of 30–40 larvae. Each data group was analysed for normal distribution using Shapiro-Wilk test. Statistical significance was determined by two-tailed unpaired t-test (parametric) with Welchs correction. *** represent p≤0.0001, ** represent p≤0.001, n.s means statistical non-significance when p≥0.05. For more statistical details and numerical data values in the scatter plot refer to S1 and S2 Data.

When the larvae sense an odour through OSNs, an activity pattern is generated at the glomerulus of AL. AL are the first processing centres of the olfactory circuit consisting of LNs, which are majorly inhibitory and only a small number of neurons are excitatory [42]. We tested naïve chemotactic response and habituation index after knock-down of ChT in LNs of AL using *GH298GAL4* driver line. *GH298GAL4* marks the majority of LNs present in the glomeruli of AL [40]. We observed that depletion of ChT by *UAS-ChT*^*RNAi1*^ and *UAS-ChT*^*RNAi2*^ in AL neurons (*GH298> UAS-ChT*^*RNAi1*^ *or UAS-ChT*^*RNAi2*^*)* significantly reduced habituation as compared to their genetic controls (*GH298*> + or *UAS-ChT*^*RNAi2*^>+) without affecting naïve chemotactic responses (Fig 5A and 5B). The control larvae recovered to the naïve levels within 30 min after stimulus removal (Fig 5C). However, we observed that the response towards odour recovered to near baseline level when ChT was knocked down with *UAS-ChT*^*RNAi1*^ but not with *UAS-ChT*^*RNAi2*^ (Fig 5C). The observed recovery obtained with *UAS-ChT*^*RNAi1*^ might be because the decrement in chemotaxis was already less upon habituation. Also, the brief dishabituating cold shock stimulus recovers the response index to the naïve levels significantly in control (*GH298*> +) but not when ChT was knocked down in *GH298*>*UAS-ChT*^*RNAi1*^ larvae (Fig 5C). The failure of group of larvae with depleted ChT to spontaneously recover and dishabituate was significantly rescued by transgenic expression of ChT on ChT^RNAi1^ background (*GH298*> *ChT*^*RNAi1*^*;UAS-ChT*) (Fig 5C). Since LNs modulate the activity of PNs and our immunostainings show ChT expression in PN presynaptic terminals, we assessed the functional role of ChT in PNs in odour processing and habituation. We used *GH146GAL4* driver to mark PNs, most of which are excitatory neurons and also have expression domain in GABAergic and octopaminergic PNs [43–45]. To determine the role of ChT in all the PNs that project from AL to MBs in olfactory habituation, we used *GH146GAL4* driver to drive *UAS-ChT*^*RNAi1*^ and *UAS-ChT*^*RNAi2*^ for knock-down of ChT (*GH146> UAS-ChT*^*RNAi1*^ *or UAS-ChT*^*RNAi2*^*)*. Knock-down of ChT in PNs did not cause any significant changes in naïve chemotactic response but caused a significant reduction in habituation index with both *UAS-ChT*^*RNAi1*^ and *UAS-ChT*^*RNAi2*^ lines as compared to the genetic controls (*GH146GAL4*>+ or *UAS-ChT*^*RNAi2*^*>+*) (Fig 5D and 5E). The habituation defect was rescued back by transgenically expressing *UAS-ChT* in larvae of genotype *GH146*> *UAS-ChT*^*RNAi1*^*;UAS-ChT* (Fig 5E). The decline in the olfactory response in the habituated larvae was recovered back to the baseline levels in the control group of larvae (*GH146*>+) over time, in the absence of a stimulus (Fig 5F) as well as on providing dishabituating cold stimulus conforming to the habituation characteristics (Fig 5F). This spontaneous recovery and dishabituation were not observed in the group of larvae with depleted ChT in genotypes *GH146*>*UAS-ChT*^*RNAi1*^ *or UAS-ChT*^*RNAi2*^ (Fig 5F).

**Fig 6:**
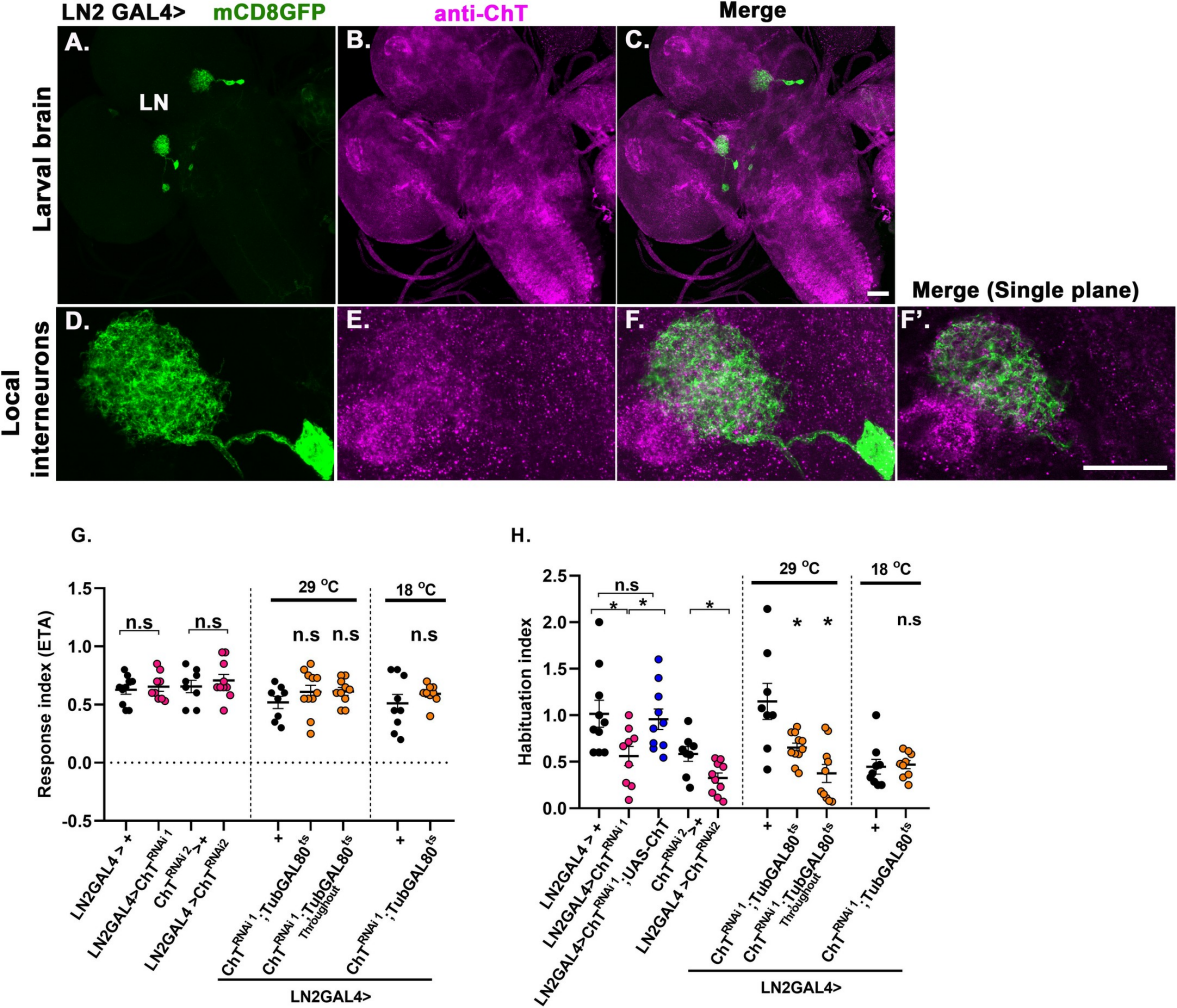
Presence of ChT in local interneurons is required for olfactory habituation but not chemotaxis. (A-C) Expression of *UAS-mCD8GFP* in local interneurons (green) driven by *LN2GAL4* and immunostained with anti-ChT (magenta) and merge, imaged with 20x, 0.9 N.A objective, scale bar 50 μm.(D-F) local interneurons imaged at higher magnification, scale bar 20 μm, imaged with 40x, 1.4N.A oil objective. (F’) represents the merged image of single plane taken from the same z-section of represented image stacks. The colocalised regions can be viewed as white areas in the merged image shown. All images are z-stacked (unless mentioned) pseudocoloured representative of 3–5 3^rd^ instar larval brains. (G) response index, (H) habituation index in genotypes: *LN2*> *UAS-ChT*^*RNAi1*^ (pink circles), *LN2> UAS-ChT*^*RNAi1*^*;UAS-ChT* (blue circles) as compared to its control *LN2*> *+* (black circles). *LN2*> *UAS-ChT*^*RNAi2*^ (pink circles) as compared to *UAS-ChT*^*RNAi2*^*>+* (black circles). *LN2>ChT*^*RNAi1*^*;TubGAL80*^*ts*^ at 29°C where ChT was knocked down only in 3^rd^ instar foraging developmental window or throughout 29°C (orange circles) as compared to *LN2*>+ (black circles). Scatter plot also shows data of genotypes *LN2>ChT*^*RNAi1*^*;TubGAL80ts* 18°C (orange circles) as compared to *LN2*>+ (black circles) at 18°C. A significant reduction in habituation index was observed while chemotaxis remains unaltered when ChT was knocked down in local interneurons. Each N represent one experiment performed with a group of 40 larvae. Each data group was analysed for normal distribution using Shapiro-Wilk test. Statistical significance was determined by two-tailed unpaired t-test (parametric) with Welchs correction. * represent p≤0.05, n.s means statistical non-significance when p≥0.05. For exact statistical details and numerical data values in the scatter plot refer to S1 and S2 Data.

To ascertain if the reduced habituation observed due to knock down of ChT in AL and PN is not a result of any developmental defect, we knocked down ChT acutely in specific 3^rd^ instar developmental window and, also, throughout development using *TubGAL80*^*ts*^ driven by GH298 and GH146 driver lines. We observed an unaltered chemotaxis when ChT was knocked down throughout the development using *TubGAL80*^*ts*^ both in AL and PN (Fig 5A and 5D). Interestingly, we observed an enhanced chemotaxis when ChT was specifically knockdown in 3^rd^ instar larvae using *GH298*Gal4 driver in genotype *GH298> UAS-ChT*^*RNAi1*^*;GAL80*^*ts*^ (Fig 5A). However, a significantly reduced habituation was observed when ChT was knocked down either in 3^rd^ instar developmental window or throughout development, both in AL and PN (Fig 5B and 5E). In our immunostaining analysis, expression domain of *GH298GAL4* was observed both in MB calyx and AL. Therefore, to delineate the effect of ChT knockdown in LN of AL from that of calyx, we used *LN2GAL4* driver line which lacks expression in projections outside AL neuropile (Fig 6A–6F’). Knockdown of ChT in genotypes LN*2*> *UAS-ChT*^*RNAi1*^ *or UAS-ChT*^*RNAi2*^ did not alter chemotaxis but significantly reduced the habituation index as compared to their genetic controls (Fig 6G and 6H). In addition, we also knocked down ChT in specific 3^rd^ instar larval developmental window using *TubGAL80*^*ts*^ and observed unaltered chemotaxis but significantly reduced habituation (Fig 6G and 6H). This experiment also explains that enhanced chemotaxis observed due to knockdown of ChT temporally in specific 3^rd^ instar window in genotypes *GH298> UAS-ChT*^*RNAi1*^*;GAL80*^*ts*^ might be due an effect arising in calyx due to knock down of ChT and not in LNs of AL. Altogether, these results clearly show that LNs of AL and PNs require ChT to facilitate olfactory habituation but is dispensable for incoming odour stimuli suppression.

Altogether, our results suggest that ChT plays a role in AL neurons, PNs, and MB intrinsic neurons to facilitate olfactory habituation. Our results highlight the dynamic engagement of ChT in first processing and higher processing centres of the larval brain to regulate responses towards stimuli that are inconsequential in nature and function in a cooperative manner to optimise synchronisation of odour perception, information transfer, and display relevant behavioural output.

### ChT is required in GABAergic neurons for both olfactory habituation and stimulus suppression but mediates only stimulus suppression via cholinergic neurons

OSNs and major class of PNs in *Drosophila* are cholinergic in nature [46]. The majority of LNs in AL are GABAergic neurons that make connections with projections both from OSNs and PNs. GABAergic connections of LNs onto the dendrites of PNs in AL are known to synchronise the PNs output [47] and regulate the changes in MB neurons further upstream in higher brain centres [48]. Habituation might result from either depression of excitatory neurotransmission [49] or potentiation of inhibitory transmission [24,50]. To understand how ChT contributes to the devaluation of inconsequential stimuli (habituation), we investigated two possible courses of action: 1) ChT might be present in GABAergic neurons and directly regulate olfactory habituation, 2) ChT might regulate ACh release from excitatory cholinergic neurons which in turn activate GABAergic neurons for inhibition.

**Fig 7:**
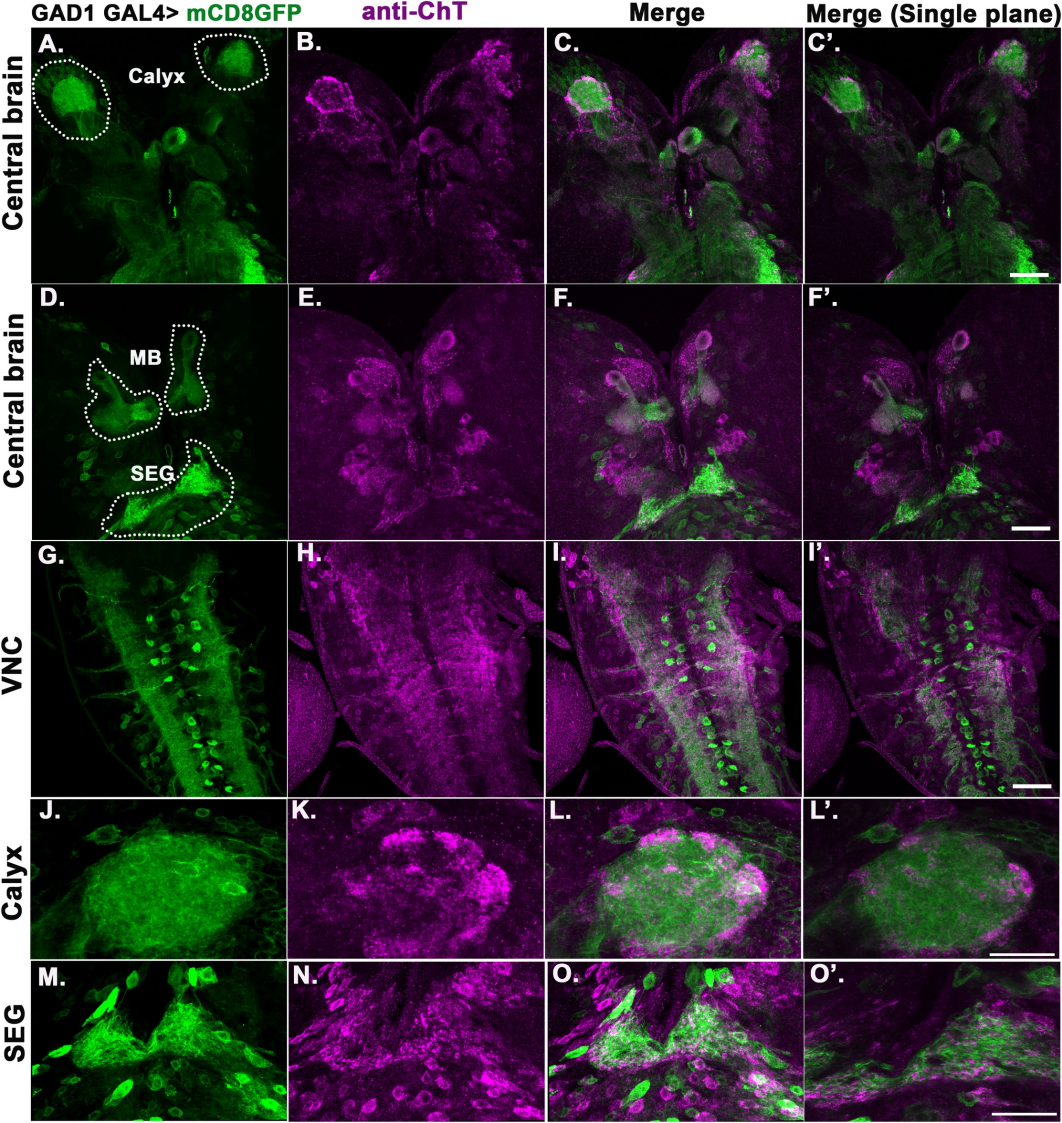
ChT is localised in GABAergic neurons of several neuropilar areas of *Drosophila* larval brain. (A-O’) Shows 3^rd^ instar larval brain. The pseudocolored images are mCD8GFP driven by *GAD1GAL4* (green), coimmunostained with anti-ChT (magenta) and merge (colocalization appears as white). (A-F’) ChT staining marks several neuropilar areas of the central brain colocalised with areas marked by *GAD1>mCD8GFP* including MB, MB calyx, and subesophageal ganglia (SEG). Respective regions of central brain are encircled with white dots and labelled, scale bar 50μm, (G-I) shows VNC, scale bar 50μm, (J-L) shows high magnification image of MB calyx, scale bar 20μm. ChT staining was observed to be colocalised with *GAD1>mCD8GFP* marked areas, specifically at the peripheral areas of calyx. Merged image shows colocalised area as white. (M-O) shows high magnification image of SEG, scale bar 20μm. ChT staining was observed to be colocalised with *GAD1>mCD8GFP* marked areas, specifically at the peripheral areas of SEG. Merged image shows colocalised area as white. (C’, F’, I’, L’ and O’) represents the merged image of single plane taken from the respective z-section of represented image stacks. All images are z-stacked (unless mentioned) representative of 3–5 3^rd^ instar larval brains.

**Fig 8:**
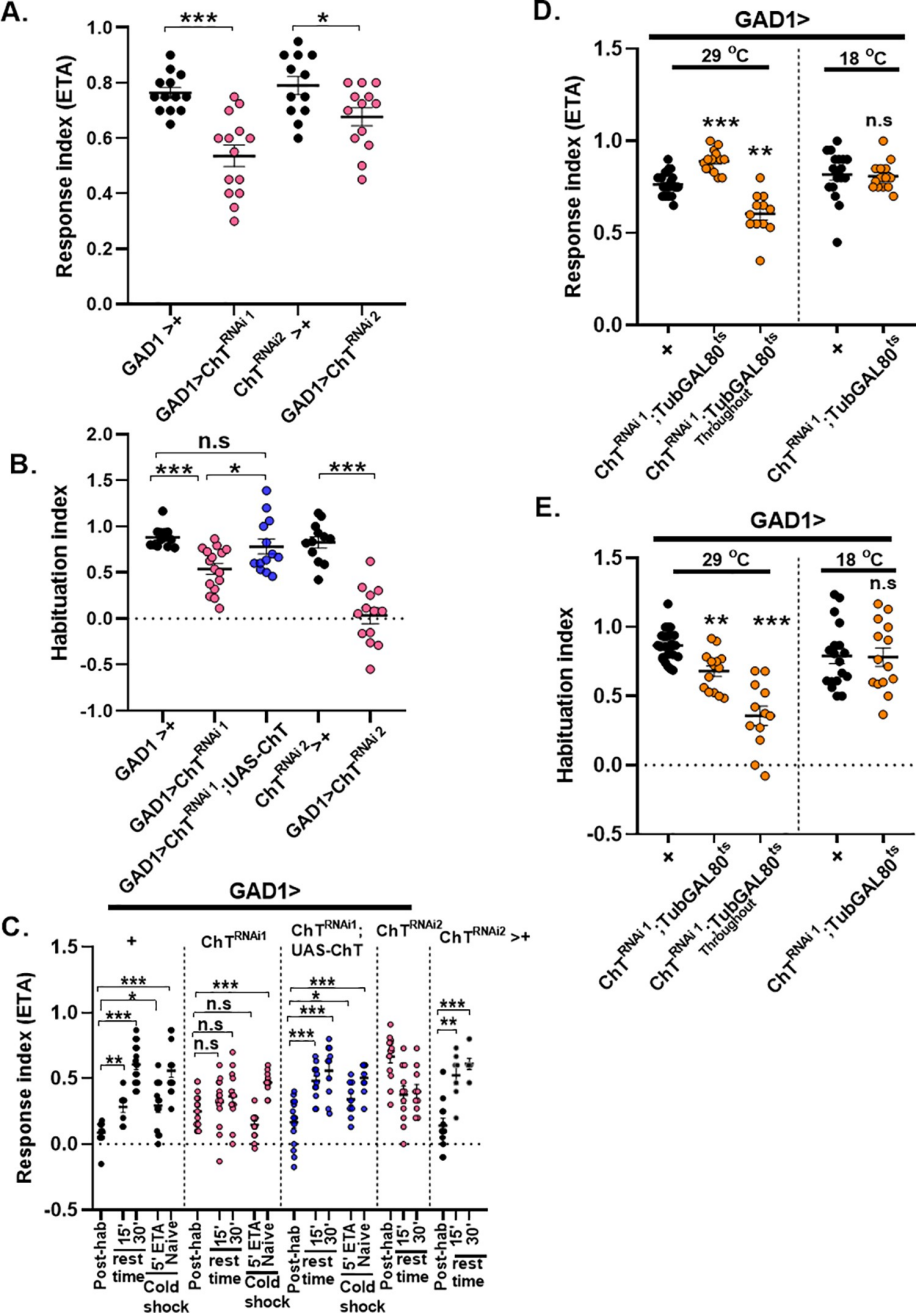
ChT is required in GABAergic neurons for olfactory habituation and chemotaxis. (A and B) shows response index and habituation index in larvae of genotypes: *GAD*> *UAS-ChT*^*RNAi1*^ (pink circles), *GAD> UAS-ChT*^*RNAi1*^*;UAS-ChT* (blue circles) as compared to its control *GAD*> *+* (black circles). *GAD*> *UAS-ChT*^*RNAi2*^ (pink circles) as compared to *UAS-ChT*^*RNAi2*^*>+* (black circles). A reduced chemotaxis and habituation was observed when ChT was knocked-down in GABAergic neurons. (C) response index following 5’ ETA exposure (post-hab), spontaneous recovery in the ETA pre-exposed larvae after 15 and 30 min rest time in the absence of stimulus, dishabituation in the ETA pre-exposed larvae presented to 1 min cold shock (5’ETA+coldshock) and naïve larvae presented to 1 min cold shock (naïve + cold shock), of the genotypes: *GAD1*> *+* (black circles), *GAD1*> *UAS-ChT*^*RNAi1*^ (pink circles), *GAD1> UAS-ChT*^*RNAi1*^*;UAS-ChT* (blue circles), *GAD1*> *UAS-ChT*^*RNAi2*^ (pink circles) and *UAS-ChT*^*RNAi2*^*>+* (black circles). Knock-down of ChT impairs spontaneous recovery and dishabituation when ChT was knock down in GABAergic neurons with *GAD1GAL4* driver. (D and E) shows response index and habituation index, respectively, in genotypes GAD1> *UAS-ChTRNAi1;TubGAL80ts* at 29°C where ChT was knocked down only in 3^rd^ instar foraging developmental window or throughout 29°C (orange circles) as compared to *GAD1*>+ (black circles). Scatter plot also shows data of genotypes *GAD1>ChT*^*RNAi1*^*;TubGAL80*^*ts*^ at 18°C (orange circles) as compared to *GAD1*>+ (black circles) at 18°C. Chemotaxis was significantly reduced, except in the condition when ChT was knocked down in 3^rd^ instar developmental window, an enhanced chemotaxis was observed. A significant reduction in habituation index was observed when ChT was knocked down in GABAergic neurons marked by *GAD1* driver line. Each N in scatter plot represent one experiment performed with a group of 30–40 larvae. Each data group was analysed for normal distribution using Shapiro-Wilk test. Statistical significance was determined by two-tailed unpaired t-test (parametric) with Welchs correction. *** represent p≤0.0001, ** represent p≤0.001, n.s means statistical non-significance when p≥0.05. For exact statistical details and numerical data values in the scatter plot refer to S1 and S2 Data.

The synthesis of the GABA neurotransmitter is catalysed by the enzyme Glutamic acid decarboxylase (GAD). *GAD1GAL4* has a predominant expression in GABAergic neurons in the fly brain [51]. We used *GAD1GAL4* to mark the putative GABAergic neurons in the larval brain by driving *UAS-mCD8GFP* and assessed with anti-ChT primary antibody if ChT is colocalised in the GABA positive regions. A colocalisation of GABA positive neurons and ChT was observed in several regions of central brain (Fig 7A–7C’), MB lobes (Fig 7D–7F’), ventral nerve cord (VNC) (Fig 7G–7I’), MB calyx (Fig 7J–7L’), and subesophageal ganglia (SEG) (Fig 7M–7O’). Further, we knocked down ChT in GABAergic neurons using *GAD1GAL4;UASmCD8GFP* genetic combination and immunostained with anti-ChT to assess ChT signal loss in GABAergic neurons. We quantified signal loss in VNC region and observed a significant reduction of ChT signals in GABAergic neurons present in VNC (S7 Fig). This suggests ChT is localised in GABAergic neurons. Although ChT is a transporter generic to ACh metabolic cycle, but interestingly we observed a major ChT immunopositive region colocalised with GABAergic terminals in several regions of larval central brain neuropile. To elucidate whether ChT is indeed required in inhibitory neurons for olfactory habituation, we knock down ChT by expressing *UAS-ChT*^*RNAi1*^ and *UAS-ChT*^*RNAi2*^ in GABAergic neurons using *GAD1*GAL4. Knock-down of ChT by both *ChT*^*RNAi1*^ and *ChT*^*RNAi2*^ significantly reduced chemotaxis as well as habituation as compared to their respective genetic controls (Fig 8A and 8B). The waning of the response towards odour stimulus was recovered back in the control group of larvae (*GAD1*GAL4>+) after 30 min rest time or by giving cold stimulus but not in ChT depleted group of larvae (*GAD1GAL4>UAS-ChT*^*RNAi1*^ and *UAS-ChT*^*RNAi2*^) (Fig 8C). The habituation ability was restored to baseline levels on transgenic expression of ChT in a *ChT*^*RNAi1*^ background in genotype GAD*1GAL4>UAS-ChT*^*RNAi1*^*;UAS-ChT* (Fig 8B and 8C). These observations suggest that ChT has a function in GABAergic neurons which is necessary for olfactory habituation. To further ascertain that the habituation defect observed due to knock-down of ChT in GABAergic neurons is an acute effect and not a developmentally driven defect, we genetically restricted the knock-down of ChT in GABAergic neurons to the shorter developmental window of 3^rd^ instar foraging larval stage using *TubGAL80*^*ts*^. Significantly enhanced chemotaxis was observed when knock-down of ChT was restricted to the specific developmental window of 3^rd^ instar foraging larvae at 29°C but not at 18°C (Fig 8D). However, when we knocked down ChT under *TubGAL80*^*ts*^ throughout development, we observed that the reduction in naïve chemotaxis (Fig 8D) was similar to our previous observation with *GAD1GAL4>UAS-ChT*^*RNAi1*^ and *GAD1GAL4>UAS-ChT*^*RNAi2*^ group of larvae (Fig 8A). The habituation index was also significantly reduced when ChT knock-down was specified to the developmental window of 3^rd^ instar foraging larvae using *TubGAL80*^*ts*^ at 29°C but not at 18°C (Fig 8E). However, the reduction was more drastic when it was knocked down throughout development (Fig 8E). These experiments allowed us to differentiate the acute effect of ChT knock-down on chemotaxis and habituation from the developmental role of these neurons. Together, these results suggest that ChT is an essential component of inhibitory GABAergic neurons and facilitates olfactory habituation directly.

**Fig 9:**
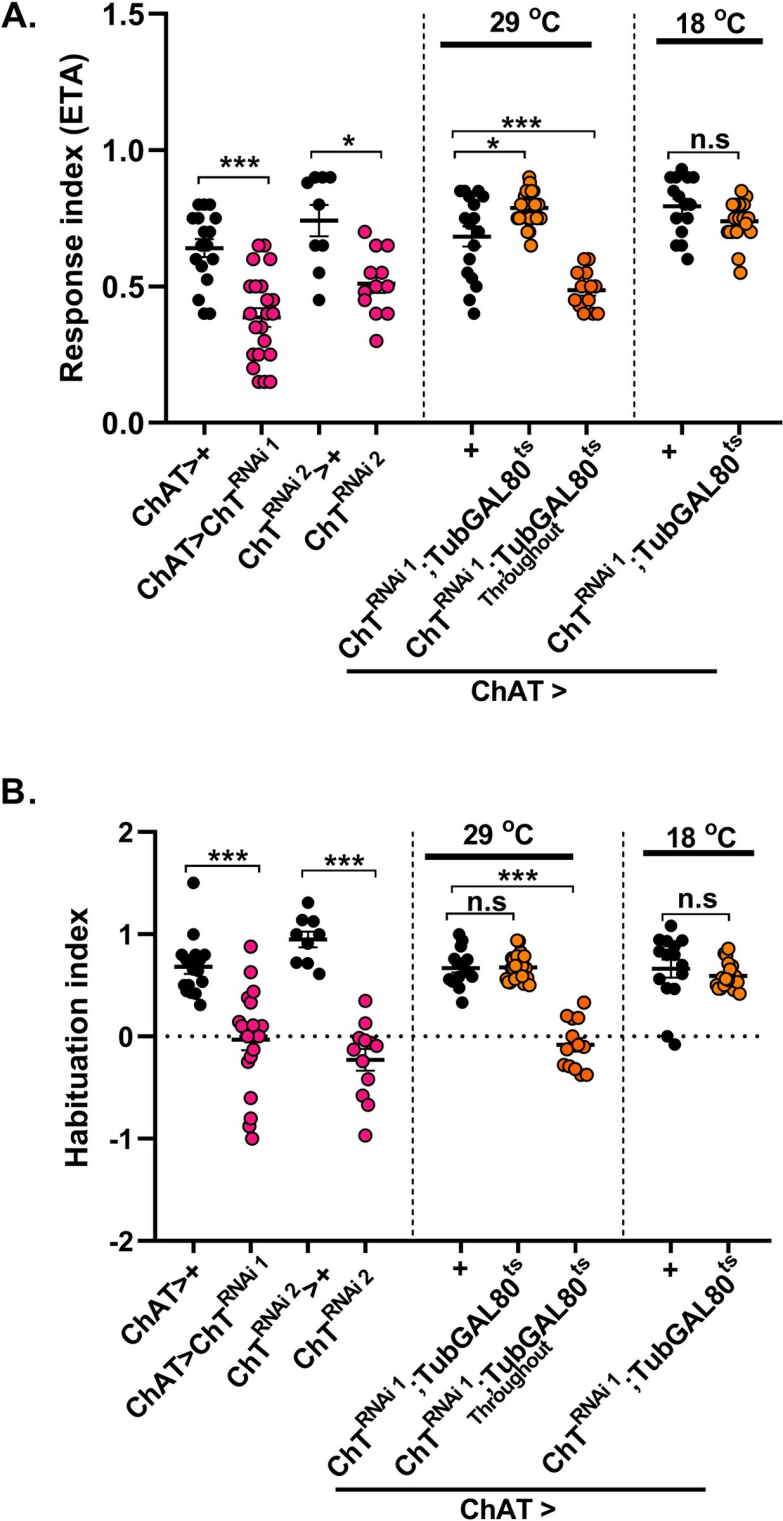
Knockdown of ChT in cholinergic neurons does not impair olfactory habituation. (A and B) shows response index and habituation index in larvae of genotypes: *ChATGAl4*>*UAS-ChT*^*RNAi1*^ (pink circles) as compared to its control *ChATGAL4*>*+* (black circles). *ChATGAl4*> *UAS-ChT*^*RNAi2*^ (pink circles) as compared to *UAS-ChT*^*RNAi2*^*>+* (black circles), *ChATGAl4* > *UAS-ChTRNAi1;TubGAL80ts* at 29°C where ChT was knocked down only in 3^rd^ instar foraging developmental window or throughout 29°C (orange circles) as compared to *ChATGAl4*>+ (black circles). Scatter plot also shows data of genotypes *ChATGAl4>ChT*^*RNAi1*^*;TubGAL80*^*ts*^ at 18°C (orange circles) as compared to *ChATGAl4*>+ (black circles) at 18°C. ChT knockdown throughout development either by expression of *UAS-ChT*^*RNAi1*^ or *UAS-ChT*^*RNAi2*^ leads to significantly reduced chemotaxis and habituation. ChT knockdown specifically at 3^rd^ instar development stage leads to enhanced chemotaxis but habituation remains unaltered. Each N in scatter plot represent one experiment performed with a group of 40 larvae. Each data group was analysed for normal distribution using Shapiro-Wilk test. Statistical significance was determined by two-tailed unpaired t-test (parametric) with Welchs correction. *** represent p≤0.0001, * represent p≤0.001, n.s means statistical non-significance when p≥0.05. For exact statistical details and numerical data values in the scatter plot refer to S1 and S2 Data.

Next, we knocked down ChT in all the cholinergic excitatory neurons using *ChATGAL4* [4] (Salvaterra and Kitamoto, 2001) which includes subsets of cholinergic LNs, PNs of the olfactory pathway and also cholinergic neurons of MBs,. Null mutant alleles of ChAT encoding genes in *Drosophila* lead to embryonic lethality and show morphological abnormalities suggesting that ACh mediated neurotransmission is essential for development [52]. Therefore, to bypass any developmental defect, we knocked down ChT in the specific foraging 3^rd^ instar larval developmental window. A drastic reduction of chemotaxis was observed when ChT was knocked down in genotypes *ChATGAL4>UAS-ChT*^*RNAi1*^ *or UAS-ChT*^*RNAi2*^ as compared to their respective controls (Fig 9A). However, enhanced chemotaxis or hypersensitivity was observed when ChT knock-down was restricted to foraging 3^rd^ instar larvae stage in genotype *ChATGAL4>UAS-ChT*^*RNAi1*^*;TubGAL80*^*ts*^ at 29°C (Fig 9A). Interestingly, we saw habituation defect only when ChT was knocked down throughout the development in genotype *ChATGAL4>UAS-ChT*^*RNAi1*^*;TubGAL80*^*ts*^ at 29°C but not when specifically knocked down during foraging 3^rd^ instar larvae (Fig 9B). The reduced chemotaxis and habituation upon knock-down of ChT throughout development might have arisen due to modulation of olfactory neural circuit development, which depends on the ACh mediated signals. ChATGAL4 driver used here contains a large 5’ flanking genomic region of *ChAT* gene which consist of the regulatory region for the most extensive spatial expression of ChAT gene [53,54]. Although unlikely, but we do not rule out the possibility if ChATGAl4 driver is missing regulatory sequences to be expressed in the neuropilar regions which are important for facilitating olfactory habituation. Perhaps due to this, we did not observe the impaired habituation.

Together, our results suggest that ChT mediates the regulation of incoming odour stimulus by a subset of both inhibitory GABAergic and excitatory cholinergic neurons but facilitates habituation only through the inhibitory GABAergic neurons. Thus, in agreement with previous published reports, our results support the involvement of inhibitory GABAergic circuit in habituation mechanism. In addition, we now demonstrate that ChT is a crucial component of this olfactory habituation pathway. The attenuated chemotaxis and habituation upon knock-down of ChT in GABAergic neurons indicate that ChT might not be unique to cholinergic neurons but may facilitate other neurotransmitter systems’ functioning too.

## Discussion

In this study, we demonstrate the role of ChT in the *Drosophila* larval olfactory circuit and higher processing centres to devalue the incoming olfactory stimuli (habituation), followed by spontaneous recovery in the absence of the stimuli and recovery upon exposure to unrelated stimuli (dishabituation) from a habituated state. We show hypersensitivity towards an attractive odour is a direct corollary of habituation defects, due to depletion of ChT in MBs. ChT function has classically been attributed to acetylcholine formation. Importantly, we report for the first time that ChT is also present in GABAergic neurons and contributes to habituation and sensory stimuli suppression. Thus, in addition to highlighting the importance of ChT in attaining habituation flexibility, this data demonstrates a relationship between upstream MB neuropilar function and downstream stimulus, mediated by ChT localised in MBs.

### ChT regulates olfactory habituation via GABAergic neurons

The requirement of control over perceived sensory stimuli is vital for an animal to acquire transition flexibility between a habituated to non-habituated state and back, enabling an animal to respond selectively to a stimulus depending on the demand. Our results show that ChT is one, if not an exclusive, regulatory molecule to facilitate this flexibility. This is supported by our observations that ChT is required for habituation, spontaneous recovery, dishabituation, and stimulus suppression. Genetic experiments demonstrate olfactory habituation in *Drosophila* as GABA mediated inhibition of PN terminals at AL which required NMDA receptors or GABA receptors on dendrites of cholinergic PNs in AL [24–50]. Intriguingly, we observed that ChT is required in GABAergic neurons to facilitate olfactory habituation, corroborating with the previous findings. Different results obtained in this study support the requirement of ChT in GABAergic neurons to regulate habituation: *Firstly*, knock down of ChT in the GABAergic neurons using specific *GAD1GAL4* driver leads to habituation defect. *Secondly*, the three kinds of neurons, namely PNs, AL, and MB neurons in the olfactory neural circuitry also have a GABAergic subset of neurons and knock down of ChT in all these neurons show habituation defect. *Thirdly*, knock down of ChT in cholinergic neurons using *ChATGAL4* does not show habituation defect. Our results with TARGET system distinguish between developmental and acute effect of ChT knock down in excitatory cholinergic and inhibitory GABAergic neurons on habituation. These results suggest that olfactory habituation is not directly modulated by cholinergic transmission rather neuronal developmental processes in the habituation circuit may be affected by cholinergic signalling. Perhaps due to this reason we observed impaired habituation and chemotaxis when ChT was constitutively knocked down in cholinergic neurons. Our interpretation of ChT expression in GABAergic neurons is based on visualisation of putative GABAergic neurons marked by *GAD1GAL4* driver, followed by assessment of ChT immunoreactivity in these locations. We do not rule out the possibility of any difference in the *GAD1-GAL4* driven expression pattern and the actual endogenous GAD1 protein distribution. Future studies are required to evaluate the detailed expression pattern of endogenous ChT and GAD1 or GABA antigens using specific antibodies. Recently, low levels of ChAT transcript were reported in GABAergic and glutamatergic neurons of specific hemilineages of larval VNC [55]. The significance, if any, of ChAT transcript in non-cholinergic neurons is unknown but raises a possibility that these neurons perhaps synthesize ACh at a specific spatial or temporal stage. Also, further investigations are required to determine the specific contribution of ChT in development of GABAergic neurons. This will be crucial in understanding impaired habituation processes underlying GABA mediated neurodevelopmental disorders.

Immunoelectron microscopic analysis of MB calyx reveals that each glomerulus comprises cholinergic nerve endings at the core encircled by numerous GABAergic terminals [56]. Therefore, MB Kenyon cells dendrites receive major excitatory inputs from cholinergic neurons and inhibitory inputs from GABAergic neurons that exist in close vicinity with each other. Such anatomy has been reported in many invertebrates such as honeybees [57], crickets [58], locusts [59], in addition to *Drosophila*. The localisation of ChT in AL and MB calyx glomerulus and the fact that both the locations have excitatory as well as inhibitory terminals in close vicinity indicate that ChT might be essential for the functioning of local transmission of neurotransmitters. We speculate that ChT is a conserved protein and its presence in both excitatory and inhibitory nerve terminals in the glomerulus of MB calyx and AL might represent a conserved phenomenon across species. More specific studies are required to understand the functional diversity of ChT in different neurotransmitter systems.

### Presence of ChT in MB neurons is required for olfactory stimulus suppression

MB lesion or ablation in *Drosophila* show elevated locomotor activity [60] and diurnal activity [61]. Conditional silencing of MB output neurons (MBON) was recently reported to show enhanced proboscis extension reflex in *Drosophila* [31]. Knock-down of dopaminergic receptors (DAMB) in MBON-1 ped>αβ also promotes yeast food-seeking behaviour in fed flies as well as odour-seeking behaviour [62]. Our finding shows that lack of ChT in MBs causes hypersensitivity towards odour corroborates with previous behavioural responses on ablation of MBs and suggests that ChT may contribute to such elicited phenotypes. We propose that ChT functions as a regulatory switch in MBs to control the perceived olfactory signals to facilitate habituation behaviour. This impetus is lost when ChT is depleted in MBs, leading to hypersensitivity. Our conclusion that ChT is required for incoming stimulus suppression for facilitation of habituation and is a MB specific phenomenon is supported by different results obtained in this study: *Firstly*, knock down of ChT in all the three kinds of MB intrinsic neurons (αβ, α’β’ and γ neurons) leads to hypersensitivity towards exposed odours as well as habituation defect. *Secondly*, knock down of ChT in AL neurons and PNs does not show hypersensitivity but show a habituation defect. *Thirdly*, perturbation of synaptic transmission from MB neurons also display hypersensitivity. Our immunostainings show a prominent localisation of ChT in MB calyx. An important converging point in our observations, shared by all the GAL4 lines (*MB247*, *C305a*, *NP1131*, *C739*, *GH298*, *GAD1*) is the presence of expression domain in calyx. We observed hypersensitivity towards odour upon knock down of ChT with all these driver lines, in addition to impaired habituation. This hypersensitivity towards odour was not observed with LN2GAL4 line, which lacks expression domain in MB calyx, neither by acute knockdown of ChT nor through its constitutive knock down. This suggests that MB calyx are an important site of action for ChT to regulate incoming odour stimuli. The effect of ChT knock down on habituation and chemotaxis in different neuron types are summarised in S1 Table.

### Proposed mechanistic model of ChT functioning at MB calyx to regulate olfactory habituation

**Fig 10:**
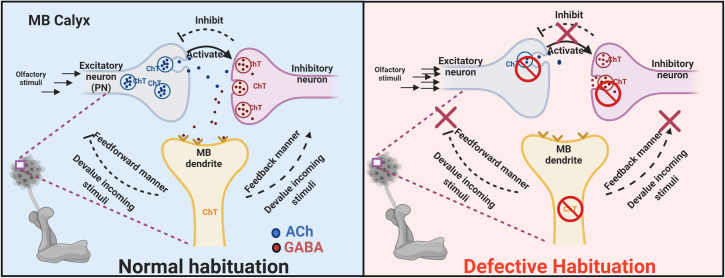
A hypothetical functional model of neuronal subsets localising ChT at glomerulus of MB calyx to regulate olfactory habituation. *Left panel*, PNs carries olfactory information to the calyx of MB and activate inhibition of MB neurons via activation of GABAergic inhibitory neurons. Both excitatory PNs and inhibitory GABAergic neurons require ChT for the forward inhibition and permit control over the MB neurons to respond to input stimuli. There are two possibilities through which ChT in MBs may further manifest control over incoming stimuli as depicted in the figure (black dashed lines): a feedback inhibition of incoming stimuli via GABAergic neurons which inturn may inhibit output from excitatory PNs or a feedforward inhibition of incoming stimuli. *Right panel*, if ChT in PNs, GABAergic or MB intrinsic neuron is depleted (shown as a red no-sign icon), the habituation is defective and the control over the incoming stimuli is lost, leading to enhanced chemotaxis towards odour, as presented in our results.

Although our results do not provide any direct evidence in an anatomical context of how ChT in MBs controls incoming stimuli, it might likely be occurring via feedback regulation from MB lobes to MB calyx or via feedforward from MB to AL. Several anatomical evidence support feedback regulation in sensory processing via connections from AL to OSNs [63], MBs to PNs [64], MB lobes to MB calyx in *Drosophila* [65], cortex to the olfactory bulb in mammals [66], cortex to visual centres in mammals [67] or feedforward regulation from MBs to AL in *Drosophila* and honeybees [68,69]. Based on our observations, we identified MB calyx as an important site for regulation of incoming odour stimulus required for olfactory habituation. Our immunostaining analysis identified ChT expression in MB calyx which has three major neuronal terminals i.e. PN terminals, Kenyon cell dendritic terminals and GABA neuron terminals. In light of these results, we propose a mechanistic model representing how ChT might mediate habituation and stimulus suppression by a synchronised activity between PN, inhibitory GABAergic neuron and MB neurons and maintains a balance between perceived stimuli and habituation (Fig 10). PN carries olfactory information to the calyx of MB and activate a feedforward inhibition of MB neurons via activation of GABAergic inhibitory neurons to induce habituation. Both PN and inhibitory GABAergic neurons requires ChT for the forward inhibition and permits control over the MB neurons to respond to input stimuli. ChT in MB might further manifest control over incoming stimuli via two pathways: it provides feedback inhibition of incoming stimuli via GABAergic neurons which in turn inhibit input from PN. ChT in MB might also permit control over incoming stimuli via feedforward inhibition of incoming stimuli. When ChT in PNs, inhibitory GABAergic neurons or MB intrinsic neurons is knocked down, the habituation is defective and the control over incoming stimuli is lost, leading to enhanced chemotaxis (or hypersensitivity). However, the potential involvement of feedback or feedforward inhibition circuit via MB in this process needs further investigation. Certainly, more precise set of experiments are warranted to confirm the functional validity of this hypothetical model.

Neuronal membranes are extremely dynamic because there is a constant need of synthesis, degradation and recycling of synaptic pools for regulating neurotransmitter secretion. Choline, imported to presynaptic terminals by ChT, is known to be acetylated for the synthesis of ACh neurotransmitter. Alternately, choline can also be phosphorylated for synthesis of phosphatidylcholine which is also one of the most abundant choline metabolites important for maintaining structural integrity of cellular membranes including neuronal membranes. We speculate that ChT is not an exclusive protein of cholinergic system but may be localised in other cell types with high need of choline, particularly neuronal cells which require it in high amounts to modulate synaptic plasticity. Here, we provide evidence that ChT is present in GABAergic neurons and plays a crucial role in facilitating olfactory habituation. Hypersensitivity has been described to be closely associated with a lack of habituating capability towards a sensory stimulus in individual with ASD [22,70,71]. ASD animal model studies and human observations also suggest dysfunction of GABA signalling [72]. Also, ChT polymorphism associated with attention deficit hyperactivity disorder (ADHD) has been reported as a key trait of Autism [73]. Further studies are warranted to investigate neural and molecular correlates of ChT in GABAergic neurons to understand GABAergic signalling related neurological disorders. It will be intriguing to also explore functional diversity of ChT in other neurotransmitter systems, which will have far reaching implications on several neurological disorders.

## Material and methods

### *Drosophila* stocks and maintenance

All *Drosophila* stocks were grown on standard BDSC formulation of fly food consisting of cornmeal-agar media supplemented with yeast, grown at 25°C unless mentioned otherwise, on a 12:12 light: dark cycle. For RNAi experiments, all fly crosses and their genetic controls were grown at 29°C. For temperature-sensitive experiments requiring GAL80, the crosses were maintained at 18°C and shifted to 29°C 24 hours before the experiments. The Gal4 drivers *MB247GAL4* (#50742), *c305aGAL4* (#30829), *Or83bGAL4* (#26818), *C739GAL4* (#7362), *GH298GAL4* (#37294), *GH146GAL4/CyoGFP* (#30026), *GAD1GAL4* (#51630), *ChATGAL4* (#6798), *TubGAL80*^*ts*^ (#7017), *UAS-mCD8GFP* (#5130) were obtained from Bloomington stock centre, Bloomington, Indiana. The *UAS-ChT*^*RNAi1*^ (#101485) line was obtained from VDRC and *UAS-*ChT^RNAi2^ (#28613) was obtained from Bloomington stock center. NP1131 (#103898) was from DGRC Kyoto stock centre, Japan. *LN2GAL4* (#NP2426) was shared by NCBS, Bangalore, India. *UAS-ChT* line was previously created and characterised by us [8]. All genetic combinations of stocks used in this study were created in the lab. For control experiments, all driver lines and responder lines were crossed with *w*^*1118*^ (+). *UAS-ChT*^*RNAi2*^ fly stock is on y^1^v^1^ background, therefore for controls, crosses with *w*^*1118*^ (+) virgins and *UAS-ChT*^*RNAi2*^ males were used.

### Antibodies and immunohistochemistry of larval brain

For immunostaining experiments, the larval brains were dissected in phosphate buffer saline (PBS), fixed in 4% paraformaldehyde followed by 5 washes with PBS+ 0.5% TritonX-100 (PBSTx) for 6 min each. The brains were incubated with 5% BSA for 40 mins and incubated overnight at 4°C with primary antibody for 16 hrs. The primary antibody was removed followed by 5 washes for 5 min each with PBSTx at room temperature. The brains were then incubated with secondary antibody for 1 hour, followed by 3 washes with PBSTx of 10 min each. The immunostained samples were mounted in vectashield, and confocal images were collected using Zeiss LSM880 microscope, Central Imaging Facility, CCMB, India. Rabbit *Drosophila* anti-ChT was used at 1:300, and mouse anti-DLG (DSHB) was used at 1:200. Optical slices were obtained in 1024x1024 pixel format using oil immersion 40x (1.4 N.A) objective. The representative images were stack projected using a maximum intensity projection module in Image J 1.52p (NIH, USA).

### ChT intensity quantification in GABAergic neuropilar regions

GABAergic neuropilar areas were marked by driving *UAS-mCD8GFP* with GAD1Gal4. For this, stable fly stock with genetic combination of *GAD1GAL4;mCD8GFP* was generated. Virgins from this fly stock was crossed with males of *w*^*1118*^ (for controls), *UAS-ChT*^*RNAi1*^ (for knock-down) or *UAS-ChT*^*RNAi1*^*; UAS-ChT* (for rescue) fly lines. The 3^rd^ instar larvae from F1 generation were dissected and immunostained with anti-ChT primary antibody and Rabbit-AlexaFluor594 secondary antibody. The confocal images were collected using 40x oil, 1.4 N.A objective in 1024 x 1024 pixel format. All imaging parameters were kept constant for scanning of all the three genotypes. All images were further quantified in the Image J. 6–10 square ROIs of 120x120 pixels were drawn in GABAergic neuropilar areas of VNC marked by mCD8GFP expression. The exact corresponding regions were marked in the second imaging channel of anti-ChT. The ChT intensity value (I_ChT_) was normalised to GFP intensity value (I_GFP_) and represented as normalised percentage intensity of ChT.

### Measurement and quantification of olfactory response index and habituation index

The assays were performed using 90 mm petridishes with 2% solid agar medium. The plates were divided into two halves and two 1.5 ml eppendorf caps were cut, autoclaved and embedded into the agar on opposite sides of the plates to place the odour. Only third instar foraging larvae (approx. 72–90 hr AEL) were used for the experiments, as they are continuously feeding and their tendency to move towards the fruity odour is higher. The assays were performed at room temperature and under red light to avoid any visual inputs. Also, all experiments were performed before noon to avoid any alterations arising due to different activity phases of larvae during the day. Forty foraging larvae were thoroughly washed and placed in the centre of the agar plate. Immediately, the odour was placed near one end of the plate and water at the opposite end. After three minutes, the number of larvae on each half of the agar plate was counted. The quantification used the midline drawn in the centre of the plate to allocate whether the larvae were on the odour side or the water side. The response towards the odour was measured by calculating the response index (R.I) and mentioned as pre-hab R.I:

R.I=(No.oflarvaetowardsodour–No.oflarvaetowardswater)÷Totalno.oflarvae

The same set of larvae were allowed to crawl on the plate while keeping lid closed for additional two minutes in the presence of the odour. Therefore, the larvae were exposed to odour for a total of 5 min for habituation. They were then placed back in the middle of the agar plate, and the response index was calculated again and mentioned as Post-hab R.I. For quantifying habituation, the Habituation index (H.I) was calculated as:

H.I=(Pre‐habR.I–Post‐habR.I)÷Pre‐habR.I

We used pure ethyl acetate (ETA), 1:100 dilution of Amyl acetate (AMA) and 1:1000 dilution of 3-Octanol for odours.

### Analysis of spontaneous recovery and dishabituation

Thirty larvae were habituated in presence of the odour for five minutes on an agar plate. Subsequently, the habituated larvae were immediately placed on a separate agar plate in the absence of odour for recovery. The response towards the odour was measured after 15, 30, and if required, 60 min. R.I was calculated as mentioned above and referred to as recovery post-hab R.I. The calculated response index after 15 or 30 min (recovery post-hab R.I) was compared with the post-hab R.I to assess for the spontaneous recovery.

For Dishabituation, the larvae were habituated for five minutes and were subsequently given a cold shock for 1 min by placing them on a pre-cooled agar plate on ice for one minute. The larvae were then kept for 2 min on agar plate without odour at room temperature to regain their movement caused due to low temperature followed by response index test. R.I towards odour was calculated as mentioned earlier and was compared with the post-hab R.I for the odour to assess for dishabituation.

### Statistical analysis

Figure alignments and statistical analysis was performed using GraphPad Prism version 8.0.0 for Windows, GraphPad Software, San Diego. All experimental groups were tested for normal distribution using Shapiro-Wilk test. The groups that passed normality test were analyzed with parametric unpaired t-test for comparison between two independent groups.Each data set was compared with their genetic controls, which is indicated separately in the individual figure legend. Data was considered significantly different if p<0.05 and represented as *** for p<0.0001, ** for p<0.001, * for p< 0.05 and n.s for non-significance when p≥0.05.

### Model

Larval anatomical schematics (Fig 4A) and hypothetical functional model for olfactory habituation (Fig 10) were prepared using BioRender (www.BioRender.com).

## Supporting information

S1 Fig RNAi mediated knock-down of ChT using *UAS-ChT^RNAi2^* shows reduction in protein levels of ChT in larval VNC. **(**A-C) Immunostained VNC with anti-Dlg (green), anti-ChT (magenta) and colocalised region shown as white in merge image in genotype *Elav;;dicer>+* compared to (D-F) with genotype *Elav;dicer>UAS-ChT*^*RNAi2*^. (G) Images of VNC immunostained with *anti-ChT* of *Elav;;dicer>+ (left)* and *Elav;;dicer>ChTRNAi2 (Right)* converted to Fire LUT map using imageJ. The scale shows the range of colours from 0-255pixel intensity. These are representative of images of 3–5 brains. Scale bar 50μm.
(TIF)

S2 Fig ChT localisation in neuropile marked by different MB GAL4 lines in *Drosophila* larval brain. Figure shows merged larval brain images of mCD8GFP (green) driven by (A) *MB247GAL4*, (B)C305aGAL4, (C) NP1131GAL4, and (D) C739GAL4 and coimmunostained with anti-ChT (magenta). The expression domains in MB regions are encircled by white dotted line. Specific regions of driver expression domain where ChT is colocalised, appears white. These are representative of images of 3–5 brains. Scale bar 50μm.
(TIF)

S3 Fig Knockdown of ChT in MB intrinsic neurons attenuates chemotaxis and habituation irrespective of the kind of odour. (A) Response index, and (B) habituation index of larvae towards amylacetate (1:100 dilution) of genotypes *NP1131>ChT*^*RNAi1*^ (pink circles, N = 14) as compared to *NP1131> +* (black circles, N = 9), *C305aGAL4>ChT*^*RNAi1*^ (pink circles, N = 6) as compared to *C305aGAL4> + (*black circles, N = 10) *and MB247GAL4>ChT*^*RNAi1*^ (Pink circles, N = 10) as compared to *MB247>+ (*black circles, N = 10). (C) Response index, and (D) habituation index towards 3-Octanol (1:1000 dilution) of genotypes *NP1131>ChTRNAi1* (Pink, N = 11) as compared to *NP1131> +* (Black, N = 11), *C305aGAL4>ChTRNAi1* (Pink, N = 10) as compared to *C305aGAL4> +*(Black, N = 9) *and MB247GAL4>ChTRNAi1* (Pink, N = 10) as compared to *MB247>+* (Black, N = 13). Each N in scatter plot represent one experiment performed with a group of 40 larvae. Each data group was analysed for normal distribution using Shapiro-Wilk test. Statistical significance was determined by two-tailed unpaired t-test (parametric) with Welchs correction. *** represent p≤0.0001, ** represent p≤0.001, n.s means statistical non-significance when p≥0.05. For exact statistical details and numerical data values in the scatter plot refer to S1 and S2 Data.
(TIF)

S4 Fig Neurotransmitter release from MB neurons is essential to regulate incoming odour stimulus and olfactory habituation. (A) Schematics showing time segments followed for the experiments. Scatter plot shows response index towards ETA, and (B) habituation index in genotypes: *C305aGAL4>UAS-Shi*^*ts*^ (orange circles) as compared to *C305aGAL4>+* (black circles), *NP1131GAL4>UAS-Shi*^*ts*^ (orange circles) as compared to *NP1131GAL4>+* (black circles), *MB247GAL4>UAS-Shi*^*ts*^ (orange circles) as compared to *MB247GAL4>+* (black circles) at 29°C and 18°C. Temporary silencing of neurotransmission by expression of temperature-sensitive mutant of Dynamin, *Shibire*, leads to significant enhancement of chemotaxis towards ETA and reduction in habituation at 29°C but not at 18°C. Each N in scatter plot represent one experiment performed with a group of 40 larvae. Each data group was analysed for normal distribution using Shapiro-Wilk test. Statistical significance was determined by two-tailed unpaired t-test (parametric) with Welchs correction. *** represent p≤0.0001, ** represent p≤0.001, n.s means statistical non-significance when p≥0.05. For exact statistical details and numerical data values in the scatter plot refer to S1 and S2 Data.
(TIF)

S5 Fig Enhanced chemotaxis and reduced habituation is an acute effect of ChT knockdown. A) Schematic shows specific time window of temperature shift for TARGET system using *TubGAL80*^*ts*^. (B) response index, and (C) habituation index of group of larvae with genotype *MB247GAL4*>*ChT*^*RNAi1*^*;TubGAL80*^*ts*^ (orange circles) compared to control *MB247GAL4*>*+* (Black circles), *NP1131GAL4*>*ChT*^*RNAi1*^*;TubGAL80*^*ts*^ (orange circles) compared to control *NP1131GAL4*>*+* (Black circles) at 29°C and 18°C, *ChT*^*RNAi1*^*;TubGAL80*^*ts*^ > + at indicated temperatures of 29°C and 18°C. (C) response index, and (D) habituation index in genotypes *C739GAL4*>*ChT*^*RNAi1*^*;TubGAL80*^*ts*^ (orange circles) where ChT is specifically knocked down in 3^rd^ instar developmental window or throughout development at 29°C compared to control *C739GAL4*>*+*. Scatter plot also shows response index and habituation index in genotypes *C739GAL4*>*ChT*^*RNAi1*^*;TubGAL80*^*ts*^ (orange circles) compared to *C739GAL4*>*+* at 18°C.Knockdown of ChT in α/β (marked by MB247 and C739) and **γ** lobe neurons (marked by NP1131) specifically in 3^rd^ instar development window shows enhanced chemotaxis and reduced habituation. Each N in scatter plot represent one experiment performed with a group of 40 larvae. Each data group was analysed for normal distribution using Shapiro-Wilk test. Statistical significance was determined by two-tailed unpaired t-test (parametric) with Welchs correction. *** represent p≤0.0001, ** represent p≤0.001, n.s means statistical non-significance when p≥0.05. For exact statistical details and numerical data values in the scatter plot refer to S1 and S2 Data.
(TIF)

S6 Fig Knock-down of ChT in olfactory sensory neurons does not affect habituation but attenuates chemotaxis. (A-C) Immunostained images of larval olfactory sensory neurons in 3^rd^ instar dissected larval brain marked by expression of mCD8GFP using *Or83bGAl4* driver in genotype *Or83bGAL4>UAS-mCD8GFP* (green), costained with anti-ChT (magenta), merge (colocalised regions of mCD8GFP and ChT appear as white). Inset shows a cropped and zoomed image of colocalised (mCD8GFP and ChT) terminal of OSNs, encircled by white dotted line. The image shown is a representative image of 3–5 brain lobes. Scale bar 50 μm (D) Response index, and (E) habituation index towards ETA of genotypes *Or83bGAL4>+* (black circles), *Or83bGAL4>ChT*^*RNAi1*^ (pink circles), *Or83bGAL4>ChT*^*RNAi1*^*;UAS-ChT* (blue circles) and *Or83bGAL4>UAS-ChT* (yellow triangles). *Or83bGAL4*>*ChT*^*RNAi1*^*;TubGAL80*^*ts*^ (orange circles) where ChT is specifically knocked down in 3^rd^ instar developmental window or throughout development at 29°C compared to control *C739GAL4*>*+*. Scatter plot also shows response index and habituation index in genotypes *Or83bGAL4*>*ChT*^*RNAi1*^*;TubGAL80*^*ts*^ (orange circles) compared to *Or83bGAL4*>*+* at 18°C. Each N in scatter plot represent one experiment performed with a group of 40 larvae. Each data group was analysed for normal distribution using Shapiro-Wilk test. Statistical significance was determined by two-tailed unpaired t-test (parametric) with Welchs correction. ** represent p≤0.001, n.s means statistical non-significance when p≥0.05. For more statistical details and numerical data values in the scatter plot refer to S1 and S2 Data.
(TIF)

S7 Fig Estimation of ChT signal reduction in GABAergic neurons by ChT^RNAi1^. Intensity quantification of anti-ChT fluorescence signal normalised to mCD8GFP intensity signal driven by GAD1GAL4. One representative image from each genotype is shown as:(A-A’) *GAD1;mCD8GFP>+*, (B-B’) *GAD1;mCD8GFP> UAS-ChT*^*RNAi1*^, (C-C’) *GAD1;mCD8GFP> UAS-ChT*^*RNAi1*^*; UAS-ChT*. All samples were imaged at similar imaging parameters. The images are shown as fire LUT map showing the scale of colours from minimum 0 pixel intensity to maximum 255 pixel intensity. The scale of intensity colours is shown on the right. (D) Bar graph shows quantification of ChT intensity signals (6–10 ROI of 120x120 pixels per brain in regions marked by *GAD1GAL4>mCD8GFP)* normalised to GFP intensity signals in the corresponding regions. Each bar represents normalised % intensity value (I_ChT_/I_GFP_) from indicated genotypes. For each genotype N = 3 brains were taken. For numerical data values representing the bar graph refer to S1 Data.
(TIF)

S1 Table Summary of effects on habituation and chemotaxis due to ChT knockdown in different neuron types. (XLSX)

S1 Data Data underlying all graphs. (XLSX)

S2 Data Sample size and Statistical details of the tests applied. (XLSX)

## References

[1] Karczmar A.G. Exploring the Vertebrate Central Cholinergic Nervous System. Springer science and Business media, LLC; 2007.

[2] Grunewald B., and Siefert P. Acetylcholine and Its Receptors in Honeybees: Involvement in Development and Impairments by Neonicotinoids. Insects. 2019;10. doi: 10.3390/insects10120420 31771114 PMC6955729

[3] Pereira L., Kratsios P., Serrano-Saiz E., Sheftel H., Mayo A.E., Hall D.H., et al. A cellular and regulatory map of the cholinergic nervous system of C. elegans. Elife. 2015;4. doi: 10.7554/eLife.12432 26705699 PMC4769160

[4] Salvaterra P.M., and Kitamoto T. Drosophila cholinergic neurons and processes visualized with Gal4/UAS-GFP. Brain Res Gene Expr Patterns. 2001; 1: 73–82. doi: 10.1016/s1567-133x(01)00011-4 15018821

[5] Tomer R., Denes A.S., Tessmar-Raible K., Arendt D. Profiling by image registration reveals common origin of annelid mushroom bodies and vertebrate pallium. Cell. 2010; 142 (5): 800–9. doi: 10.1016/j.cell.2010.07.043 20813265

[6] Barnstedt O., Owald D., Felsenberg J., Brain R., Moszynski J.P., Talbot C.B., et al. Memory-Relevant Mushroom Body Output Synapses Are Cholinergic. Neuron. 2016;89: 1237–1247. doi: 10.1016/j.neuron.2016.02.015 26948892 PMC4819445

[7] Croset V., Treiber C.D., and Waddell S. Cellular diversity in the Drosophila midbrain revealed by single-cell transcriptomics. Elife. 2018;7. doi: 10.7554/eLife.34550 29671739 PMC5927767

[8] Hamid R., Hajirnis N., Kushwaha S., Saleem S., Kumar V., and Mishra R.K. Drosophila Choline transporter non-canonically regulates pupal eclosion and NMJ integrity through a neuronal subset of mushroom body. Dev Biol. 2019; 446: 80–93. doi: 10.1016/j.ydbio.2018.12.006 30529058

[9] Keene A.C., and Waddell S. Drosophila olfactory memory: single genes to complex neural circuits. Nat Rev Neurosci. 2007;8: 341–354. doi: 10.1038/nrn2098 17453015

[10] Duerr J.S., and Quinn W.G. Three Drosophila mutations that block associative learning also affect habituation and sensitization. Proc Natl Acad Sci U S A. 1982;79: 3646–3650. doi: 10.1073/pnas.79.11.3646 6808513 PMC346480

[11] Engel J.E., and Wu C.F. Neurogenetic approaches to habituation and dishabituation in Drosophila. Neurobiol Learn Mem. 2009;92: 166–175. doi: 10.1016/j.nlm.2008.08.003 18765288 PMC2730516

[12] Acevedo S.F., Froudarakis E.I., Tsiorva A.A., and Skoulakis E.M. Distinct neuronal circuits mediate experience-dependent, non-associative osmotactic responses in Drosophila. Mol Cell Neurosci. 2007;34: 378–389. doi: 10.1016/j.mcn.2006.11.011 17197197

[13] Cho W., Heberlein U., and Wolf F.W. Habituation of an odorant-induced startle response in Drosophila. Genes Brain Behav. 2004;3: 127–137. doi: 10.1111/j.1601-183x.2004.00061.x 15140008

[14] Roussou I.G., Papanikolopoulou K., Savakis C., and Skoulakis E.M.C. Drosophila Bruton’s Tyrosine Kinase Regulates Habituation Latency and Facilitation in Distinct Mushroom Body Neurons. J Neurosci. 2019; 39: 8730–8743. doi: 10.1523/JNEUROSCI.0633-19.2019 31530645 PMC6820202

[15] Eisenstein EM, Eisenstein D, Smith, James C. The evolutionary significance of habituation and sensitization across phylogeny: A behavioral homeostasis model. Integ Physiol Behav Sci. 2001;36(4): 251–65.

[16] Semelidou O., Acevedo S.F., and Skoulakis E.M. Temporally specific engagement of distinct neuronal circuits regulating olfactory habituation in Drosophila. Elife. 2018;7. doi: 10.7554/eLife.39569 30576281 PMC6303106

[17] Ramaswami M. Network plasticity in adaptive filtering and behavioral habituation. Neuron. 2014;82: 1216–1229. doi: 10.1016/j.neuron.2014.04.035 24945768

[18] Twick I., Lee J.A., and Ramaswami M. Olfactory habituation in Drosophila-odor encoding and its plasticity in the antennal lobe. Prog Brain Res. 2014;208: 3–38. doi: 10.1016/B978-0-444-63350-7.00001-2 24767477

[19] Foss-Feig J.H., Tadin D., Schauder K.B., and Cascio C.J. A substantial and unexpected enhancement of motion perception in autism. J Neurosci. 2013;33: 8243–8249. doi: 10.1523/JNEUROSCI.1608-12.2013 23658163 PMC3726259

[20] Gomot M., Giard M.H., Adrien J.L., Barthelemy C., and Bruneau N. Hypersensitivity to acoustic change in children with autism: electrophysiological evidence of left frontal cortex dysfunctioning. Psychophysiology. 2002;39: 577–584. doi: 10.1017.S0048577202394058 12236323 10.1017/S0048577202394058

[21] Hudac C.M., DesChamps T.D., Arnett A.B., Cairney B.E., Ma R., Webb S.J., et al. Early enhanced processing and delayed habituation to deviance sounds in autism spectrum disorder. Brain Cogn. 2018;123: 110–119. doi: 10.1016/j.bandc.2018.03.004 29550506 PMC5893357

[22] Kleinhans N.M., Johnson L.C., Richards T., Mahurin R., Greenson J., Dawson G., et al. Reduced neural habituation in the amygdala and social impairments in autism spectrum disorders. Am J Psychiatry. 2009;166: 467–475. doi: 10.1176/appi.ajp.2008.07101681 19223437

[23] Fenckova M., Blok L.E.R., Asztalos L., Goodman D.P., Cizek P., Singgih E.L., et al. Habituation Learning Is a Widely Affected Mechanism in Drosophila Models of Intellectual Disability and Autism Spectrum Disorders. Biol Psychiatry. 2019;86: 294–305. doi: 10.1016/j.biopsych.2019.04.029 31272685 PMC7053436

[24] Das S., Sadanandappa M.K., Dervan A., Larkin A., Lee J.A., Sudhakaran I.P., et al. Plasticity of local GABAergic interneurons drives olfactory habituation. Proc Natl Acad Sci U S A. 2011;108: E646–654. doi: 10.1073/pnas.1106411108 21795607 PMC3169145

[25] Larkin A., Karak S., Priya R., Das A., Ayyub C., Ito K., et al. Central synaptic mechanisms underlie short-term olfactory habituation in Drosophila larvae. Learn Mem. 2010; 17: 645–653. doi: 10.1101/lm.1839010 21106688

[26] Khurana S., and Siddiqi O. Olfactory responses of Drosophila larvae. Chem Senses. 2013;38: 315–323. doi: 10.1093/chemse/bjs144 23363465

[27] Monte P., Woodard C., Ayer R., Lilly M., Sun H., Carlson J. Characterization of the larval olfactory response in Drosophila and its genetic basis. Behav Genet. 1989;19 267–283. doi: 10.1007/BF01065910 2497723

[28] Rodrigues V. Olfactory behavior of Drosophila melanogaster. In Siddiqi O.Babu P., Hall L. M., and Hall J. C. (eds.), *The Development and Neurobiology of Drosophila*,Plenum, New York; 1980. pp. 361–371. doi: 10.1007/978-1-4684-7968-3_26 6779801

[29] Aceves-Pina E., and Quinn W. Learning in normal and mutant *Drosophila* larvae. *Science*. 1979;206: 93–96. doi: 10.1126/science.206.4414.93 17812455

[30] Rankin C.H., Abrams T., Barry R.J., Bhatnagar S., Clayton D.F., Colombo J., et al. Habituation revisited: an updated and revised description of the behavioral characteristics of habituation. Neurobiol Learn Mem. 2009;92: 135–138. doi: 10.1016/j.nlm.2008.09.012 18854219 PMC2754195

[31] Chia J., and Scott K. Activation of specific mushroom body output neurons inhibits proboscis extension and sucrose consumption. PLoS One. 2020; 15:e0223034. doi: 10.1371/journal.pone.0223034 31990947 PMC6986700

[32] Krashes M.J., Keene A.C., Leung B., Armstrong J.D., and Waddell S. Sequential use of mushroom body neuron subsets during drosophila odor memory processing. Neuron. 2007;53: 103–115. doi: 10.1016/j.neuron.2006.11.021 17196534 PMC1828290

[33] Brand A.H., and Perrimon N. Targeted gene expression as a means of altering cell fates and generating dominant phenotypes. Development. 1993;118: 401–415. 8223268 10.1242/dev.118.2.401

[34] Kitamoto T. Conditional modification of behaviour in Drosophila by targeted expression of a temperature-sensitive shibire allele in defined neurons. J Neurobiol. 2001;47:81–92. doi: 10.1002/neu.1018 11291099

[35] McGuire S.E., Mao Z., and Davis R.L. Spatiotemporal gene expression targeting with the TARGET and gene-switch systems in Drosophila. Sci STKE. 2004: pl6. doi: 10.1126/stke.2202004pl6 14970377

[36] Aso Y., Hattori D., Yu Y., Johnston R. M., Iyer N. A., Ngo T. T., et al. The neuronal architecture of the mushroom body provides a logic for associative learning. Elife 2014:3. doi: 10.7554/eLife.04577 25535793 PMC4273437

[37] Python F., and Stocker R.F. Immunoreactivity against choline acetyltransferase, gamma-aminobutyric acid, histamine, octopamine, and serotonin in the larval chemosensory system of Dosophila melanogaster. J Comp Neurol. 2002;453: 157–167. doi: 10.1002/cne.10383 12373781

[38] Larsson M.C., Domingos A.I., Jones W.D., Chiappe M.E., Amrein H., and Vosshall L.B. Or83b encodes a broadly expressed odorant receptor essential for Drosophila olfaction. Neuron. 2004;43: 703–714. doi: 10.1016/j.neuron.2004.08.019 15339651

[39] Masse N.Y., Turner G.C., Jefferis G.S. Olfactory information processing in Drosophila. Curr. Biol. 2009;16: R700–13. doi: 10.1016/j.cub.2009.06.026 19706282

[40] Tanaka N.K., Endo K., and Ito K. Organization of antennal lobe-associated neurons in adult Drosophila melanogaster brain. J Comp Neurol. 2012;520: 4067–4130. doi: 10.1002/cne.23142 22592945

[41] Masuda-Nakagawa L.M., Gendre N., O’Kane C.J., and Stocker R.F. Localized olfactory representation in mushroom bodies of Drosophila larvae. Proc Natl Acad Sci U S A. 2009;106: 10314–10319. doi: 10.1073/pnas.0900178106 19502424 PMC2700900

[42] Silbering A.F., Okada R., Ito K., Galizia C.G. Olfactory information processing in the Drosophila antennal lobe: anything goes wrong? J Neurosci. 2008;28: 13075–87. doi: 10.1523/JNEUROSCI.2973-08.2008 19052198 PMC6671615

[43] Liang L., Li Y., Potter C.J., Yizhar O., Deisseroth K., Tsien R.W., et al. GABAergic projection neurons route selective olfactory inputs to specific higher-order neurons. Neuron. 2013; 79: 917–31. doi: 10.1016/j.neuron.2013.06.014 24012005 PMC3838762

[44] Liu X., Davis R.L. The GABAergic anterior paired lateral neuron suppresses and is suppressed by olfactory learning. Nat Neurosc. 2009;12: 53–59. doi: 10.1038/nn.2235 19043409 PMC2680707

[45] Wu C.L., Shih M.F., Lee P.T., Chiang A.S. An octopamine-mushroom body circuit modulates the formation of anesthesia-resistant memory in Drosophila. Curr. Biol. 2013;23: 2346–2354 doi: 10.1016/j.cub.2013.09.056 24239122

[46] Yasuyama K., and Salvaterra P.M. Localization of choline acetyltransferase-expressing neurons in Drosophila nervous system. Microsc Res Tech. 1999;45: 65–79. doi: 10.1002/(SICI)1097-0029(19990415)45:2&lt;65::AID-JEMT2&gt;3.0.CO;2-0 10332725

[47] Wilson R.I., and Laurent G. Role of GABAergic inhibition in shaping odor-evoked spatiotemporal patterns in the Drosophila antennal lobe. J Neurosci. 2005;25: 9069–9079. doi: 10.1523/JNEUROSCI.2070-05.2005 16207866 PMC6725763

[48] Laurent G., MacLeod K., Stopfer M., and Wehr M. Spatiotemporal structure of olfactory inputs to the mushroom bodies. Learn Mem. 1998;5: 124–132. 10454377 PMC311249

[49] Glanzman D.L. Habituation in Aplysia: the Cheshire cat of neurobiology. Neurobiol Learn Mem. 2009;92: 147–154. doi: 10.1016/j.nlm.2009.03.005 19332142

[50] Paranjpe P., Rodrigues V., VijayRaghavan K., and Ramaswami M. Gustatory habituation in Drosophila relies on rutabaga (adenylate cyclase)-dependent plasticity of GABAergic inhibitory neurons. Learn Mem. 2012;19: 627–635. doi: 10.1101/lm.026641.112 23169996

[51] Okada R., Awasaki T., and Ito K. Gamma-aminobutyric acid (GABA)-mediated neural connections in the Drosophila antennal lobe. J Comp Neurol. 2009;514: 74–91. doi: 10.1002/cne.21971 19260068

[52] Kitamoto T., Xie X., Wu C.F., and Salvaterra P.M. Isolation and characterization of mutants for the vesicular acetylcholine transporter gene in Drosophila melanogaster. J Neurobiol 2000; 42: 161–171. 10640324

[53] Kitamoto T., Ikeda K., Salvaterra P.M. Analysis of cis-regulatory elements in the 5’ flanking region of the Drosophila melanogaster choline acetyltransferase gene. J.Neurosci. 1992;12: 1628–1639. doi: 10.1523/JNEUROSCI.12-05-01628.1992 1374460 PMC6575896

[54] Kitamoto T., Salvaterra P.M. Developmental regulatory elements in the 5’ flanking DNA of the Drosophila choline acetyltransferase gene. Roux Arch. Dev. Biol. 1993;202: 159–169. 10.1007/BF00365306 28305993

[55] Lacin H., Chen H.M, Long X., Singer R.H., Lee T., Truman J.W. Neurotransmitter identity is acquired in a lineage-restricted manner in the Drosophila CNS. Elife. 2019;8. 10.7554/eLife.43701 PMC6504232 30912745

[56] Yasuyama K., Meinertzhagen I.A., and Schurmann F.W. Synaptic organization of the mushroom body calyx in Drosophila melanogaster. J Comp Neurol. 2002;445: 211–226. doi: 10.1002/cne.10155 11920702

[57] Ganeshina O., and Menzel R. GABA-immunoreactive neurons in the mushroom bodies of the honeybee: an electron microscopic study. J Comp Neurol. 2001;437: 335–349. doi: 10.1002/cne.1287 11494260

[58] Schurmann F.W., Frambach I., and Elekes K. GABAergic synaptic connections in mushroom bodies of insect brains. Acta Biol Hung. 2008;59 Suppl: 173–181. doi: 10.1556/ABiol.59.2008.Suppl.26 18652390

[59] Leitch B., and Laurent G. GABAergic synapses in the antennal lobe and mushroom body of the locust olfactory system. J Comp Neurol. 1996;372: 487–514. doi: 10.1002/(SICI)1096-9861(19960902)372:4&lt;487::AID-CNE1&gt;3.0.CO;2-0 8876449

[60] Heisenberg M., Borst A., Wagner S., and Byers D. Drosophila mushroom body mutants are deficient in olfactory learning. J Neurogenet. 1985;2: 1–30. doi: 10.3109/01677068509100140 4020527

[61] Martin J.R., Ernst R., and Heisenberg M. Mushroom bodies suppress locomotor activity in Drosophila melanogaster. Learn Mem. 1998;5: 179–191. 10454382 PMC311252

[62] Tsao C.H., Chen C.C., Lin C.H., Yang H.Y., and Lin S. Drosophila mushroom bodies integrate hunger and satiety signals to control innate food-seeking behavior. Elife. 2018;7. doi: 10.7554/eLife.35264 29547121 PMC5910021

[63] Olsen S.R., and Wilson R.I. Lateral presynaptic inhibition mediates gain control in an olfactory circuit. Nature. 2008;452: 956–960. doi: 10.1038/nature06864 18344978 PMC2824883

[64] Tanaka N.K., Ito K., and Stopfer M. Odor-evoked neural oscillations in Drosophila are mediated by widely branching interneurons. J Neurosci. 2009;29: 8595–8603. doi: 10.1523/JNEUROSCI.1455-09.2009 19571150 PMC2753235

[65] Masuda-Nakagawa L.M., Ito K., Awasaki T., and O’Kane C.J. A single GABAergic neuron mediates feedback of odor-evoked signals in the mushroom body of larval Drosophila. Front Neural Circuits. 2014;8: 35. doi: 10.3389/fncir.2014.00035 24782716 PMC3988396

[66] Balu R., Pressler R.T., and Strowbridge B.W. Multiple modes of synaptic excitation of olfactory bulb granule cells. J Neurosci. 2007;27: 5621–5632. doi: 10.1523/JNEUROSCI.4630-06.2007 17522307 PMC6672747

[67] Tiesinga P., Fellous J.M., and Sejnowski T.J. Regulation of spike timing in visual cortical circuits. Nat Rev Neurosci. 2008;9: 97–107. doi: 10.1038/nrn2315 18200026 PMC2868969

[68] Hu A., Zhang W., and Wang Z. Functional feedback from mushroom bodies to antennal lobes in the Drosophila olfactory pathway. Proc Natl Acad Sci U S A. 2010;107: 10262–10267. doi: 10.1073/pnas.0914912107 20479249 PMC2890443

[69] Kirschner S., Kleineidam C.J., Zube C., Rybak J., Grunewald B., and Rossler W. Dual olfactory pathway in the honeybee, Apis mellifera. J Comp Neurol. 2006;499: 933–952. doi: 10.1002/cne.21158 17072827

[70] Guiraud J.A., Kushnerenko E., Tomalski P., Davies K., Ribeiro H., Johnson M.H., et al. Differential habituation to repeated sounds in infants at high risk for autism. Neuroreport. 2011;22: 845–849. doi: 10.1097/WNR.0b013e32834c0bec 21934535

[71] Webb S.J., Jones E.J., Merkle K., Namkung J., Toth K., Greenson J., et al. Toddlers with elevated autism symptoms show slowed habituation to faces. Child Neuropsychol. 2010;16: 255–278. doi: 10.1080/09297041003601454 20301009 PMC2989718

[72] Cellot G., and Cherubini E. GABAergic signaling as therapeutic target for autism spectrum disorders. Front Pediatr. 2014;2: 70. doi: 10.3389/fped.2014.00070 25072038 PMC4085902

[73] English B.A., Hahn M.K., Gizer I.R., Mazei-Robison M., Steele A., Kurnik D.M., et al. Choline transporter gene variation is associated with attention-deficit hyperactivity disorder. J Neurodev Disord. 2009;1: 252–263. doi: 10.1007/s11689-009-9033-8 21547719 PMC3164006

