# Supplementary figures and images for: Choline Transporter regulates olfactory habituation via a neuronal triad of excitatory, inhibitory and mushroom body neurons
Source: PLoS Genet. 2021 Dec 16;17(12):e1009938. doi: 10.1371/journal.pgen.1009938 (PMC8675691; doi:10.1371/journal.pgen.1009938)

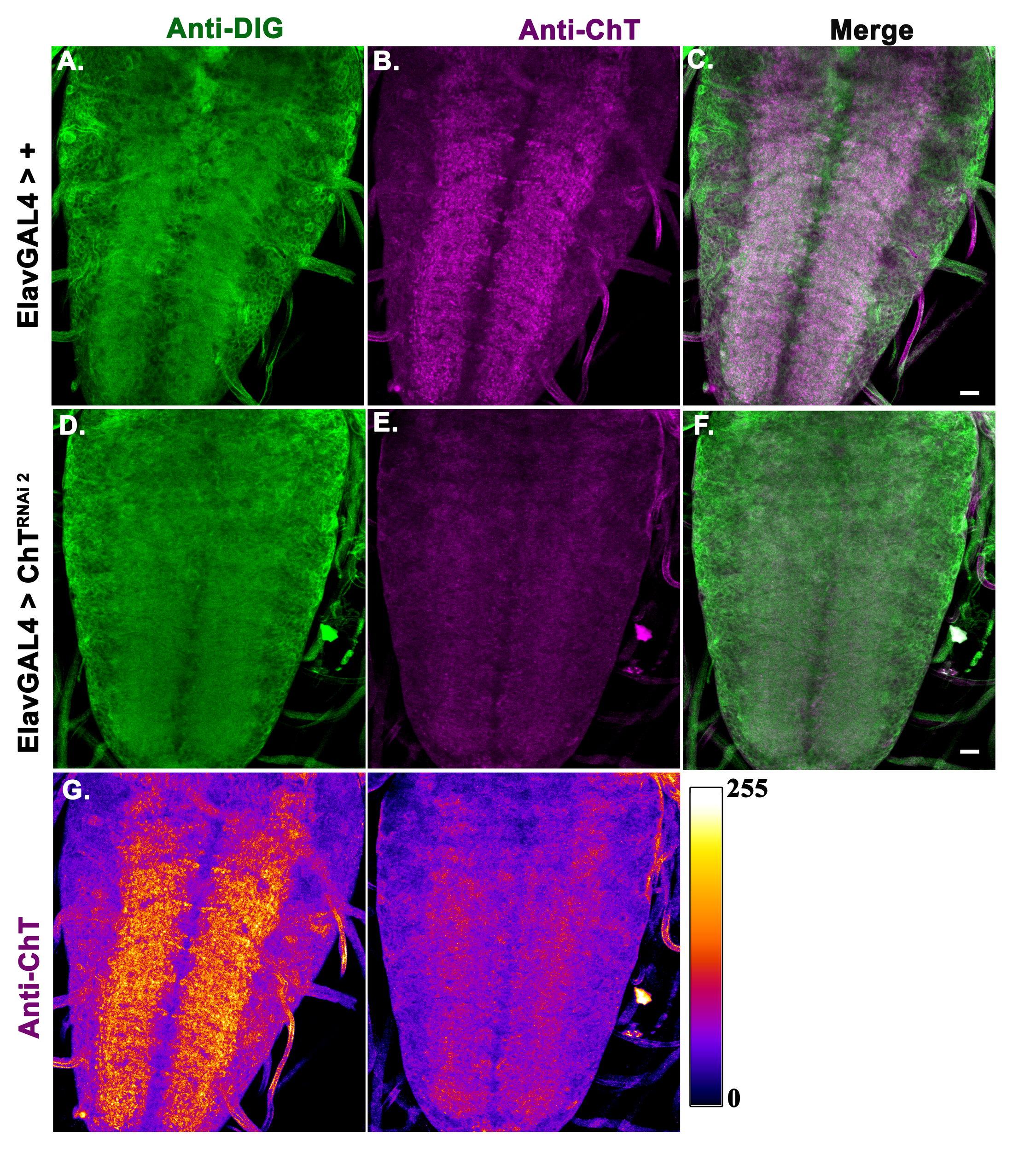

Supplement: S1 Fig — (A-C) Immunostained VNC with anti-Dlg (green), anti-ChT (magenta) and colocalised region shown as white in merge image in genotype Elav;;dicer>+ compared to (D-F) with genotype Elav;dicer>UAS-ChTRNAi2. (G) Images of VNC immunostained with anti-ChT of Elav;;dicer>+ (left) and Elav;;dicer>ChTRNAi2 (Right) converted to Fire LUT map using imageJ. The scale shows the range of colours from 0-255pixel intensity. These are representative of images of 3–5 brains. Scale bar 50μm. (TIF) [file pgen.1009938.s001.tif]

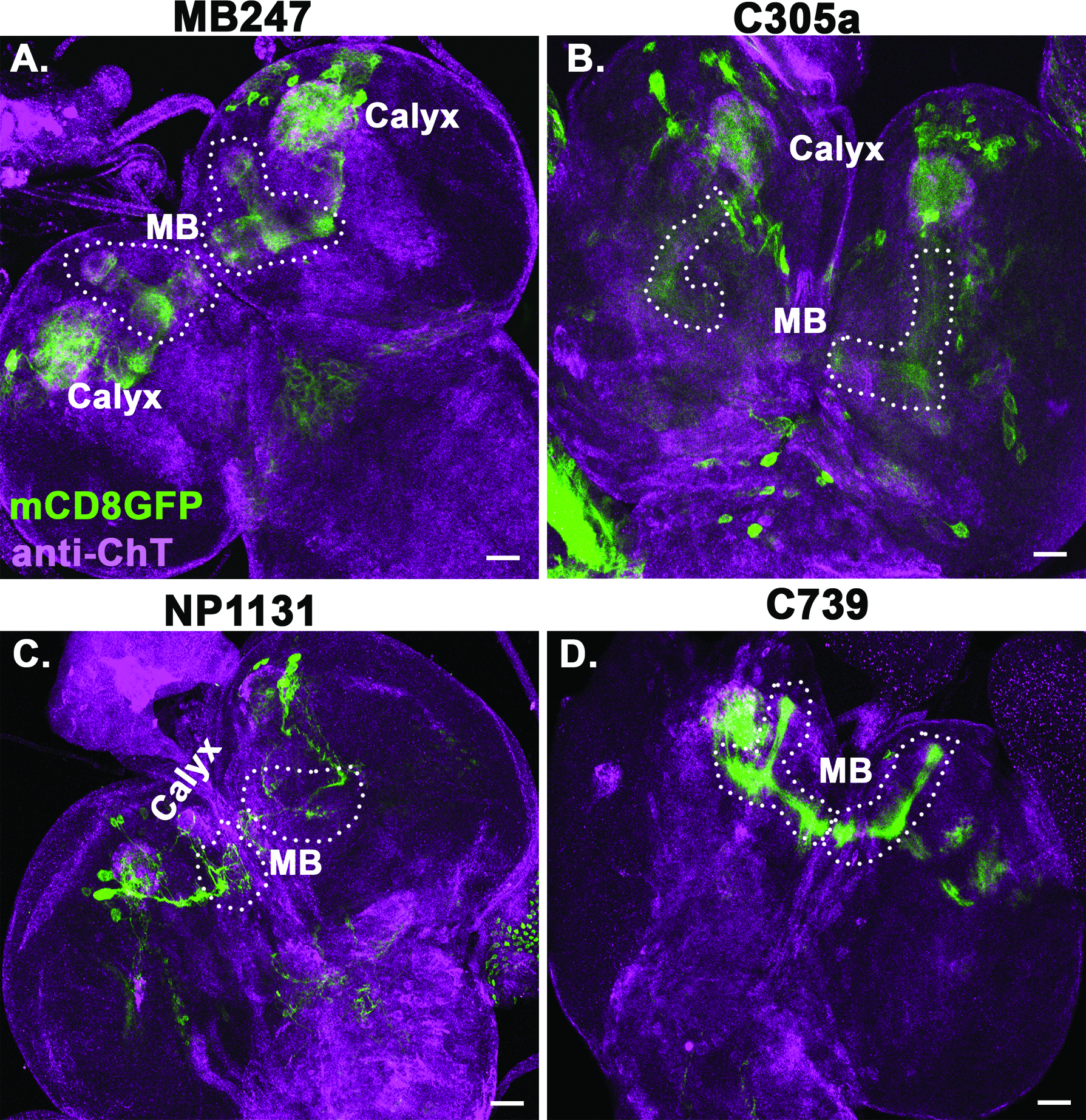

Supplement: S2 Fig — Figure shows merged larval brain images of mCD8GFP (green) driven by (A) MB247GAL4, (B)C305aGAL4, (C) NP1131GAL4, and (D) C739GAL4 and coimmunostained with anti-ChT (magenta). The expression domains in MB regions are encircled by white dotted line. Specific regions of driver expression domain where ChT is colocalised, appears white. These are representative of images of 3–5 brains. Scale bar 50μm. (TIF) [file pgen.1009938.s002.tif]

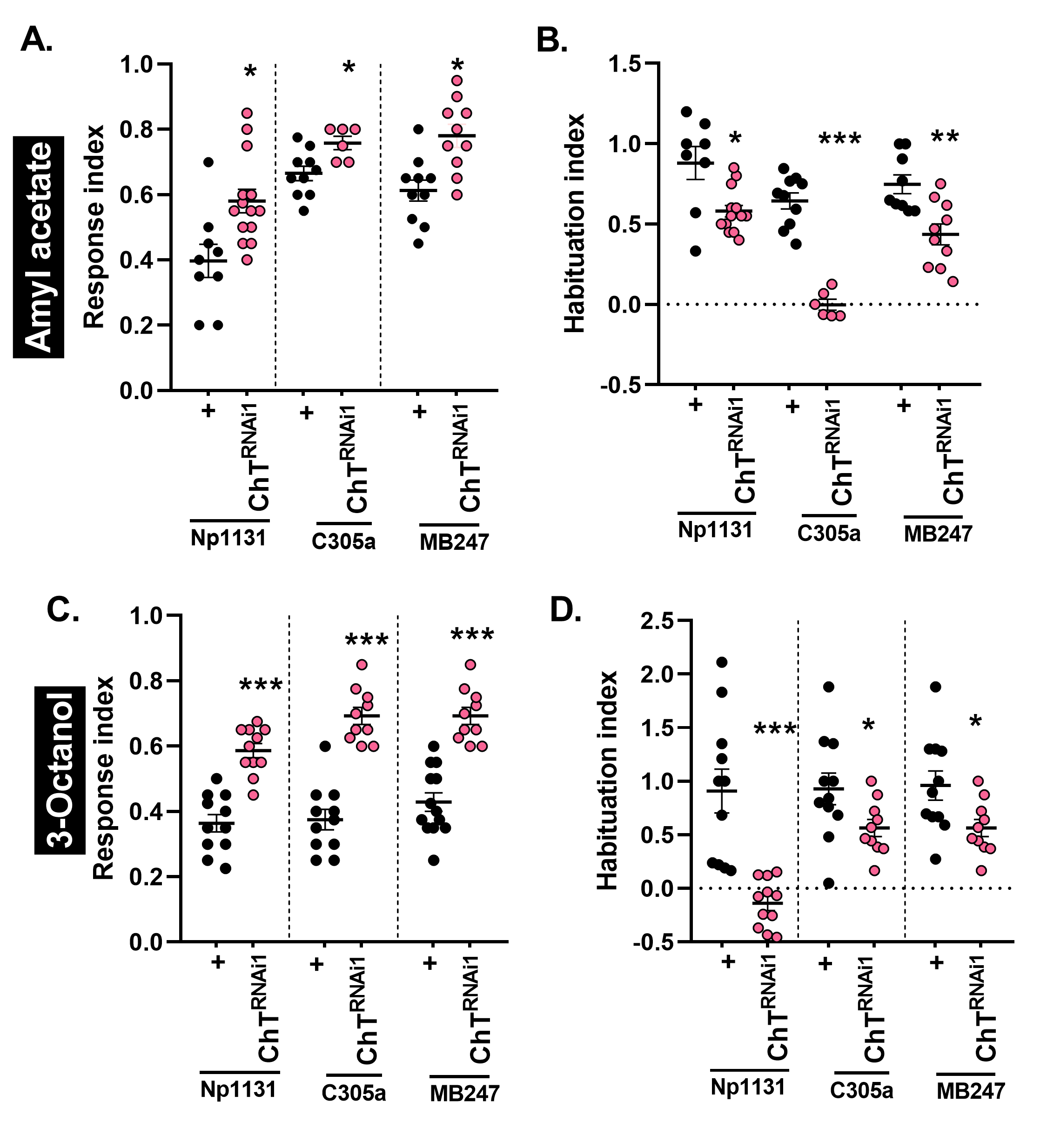

Supplement: S3 Fig — (A) Response index, and (B) habituation index of larvae towards amylacetate (1:100 dilution) of genotypes NP1131>ChTRNAi1 (pink circles, N = 14) as compared to NP1131> + (black circles, N = 9), C305aGAL4>ChTRNAi1 (pink circles, N = 6) as compared to C305aGAL4> + (black circles, N = 10) and MB247GAL4>ChTRNAi1 (Pink circles, N = 10) as compared to MB247>+ (black circles, N = 10). (C) Response index, and (D) habituation index towards 3-Octanol (1:1000 dilution) of genotypes NP1131>ChTRNAi1 (Pink, N = 11) as compared to NP1131> + (Black, N = 11), C305aGAL4>ChTRNAi1 (Pink, N = 10) as compared to C305aGAL4> +(Black, N = 9) and MB247GAL4>ChTRNAi1 (Pink, N = 10) as compared to MB247>+ (Black, N = 13). Each N in scatter plot represent one experiment performed with a group of 40 larvae. Each data group was analysed for normal distribution using Shapiro-Wilk test. Statistical significance was determined by two-tailed unpaired t-test (parametric) with Welchs correction. *** represent p≤0.0001, ** represent p≤0.001, n.s means statistical non-significance when p≥0.05. For exact statistical details and numerical data values in the scatter plot refer to S1 and S2 Data. (TIF) [file pgen.1009938.s003.tif]

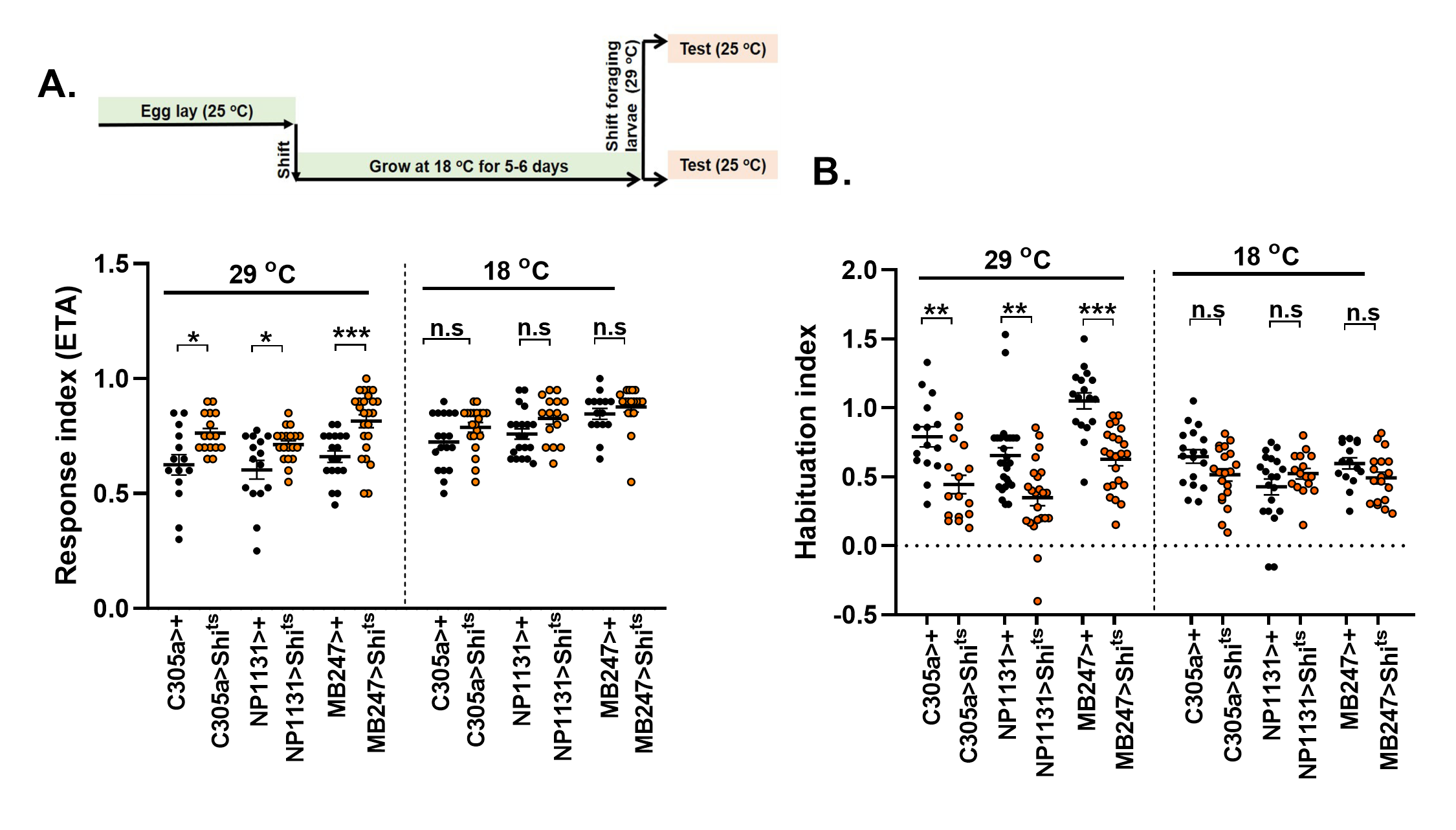

Supplement: S4 Fig — (A) Schematics showing time segments followed for the experiments. Scatter plot shows response index towards ETA, and (B) habituation index in genotypes: C305aGAL4>UAS-Shits (orange circles) as compared to C305aGAL4>+ (black circles), NP1131GAL4>UAS-Shits (orange circles) as compared to NP1131GAL4>+ (black circles), MB247GAL4>UAS-Shits (orange circles) as compared to MB247GAL4>+ (black circles) at 29°C and 18°C. Temporary silencing of neurotransmission by expression of temperature-sensitive mutant of Dynamin, Shibire, leads to significant enhancement of chemotaxis towards ETA and reduction in habituation at 29°C but not at 18°C. Each N in scatter plot represent one experiment performed with a group of 40 larvae. Each data group was analysed for normal distribution using Shapiro-Wilk test. Statistical significance was determined by two-tailed unpaired t-test (parametric) with Welchs correction. *** represent p≤0.0001, ** represent p≤0.001, n.s means statistical non-significance when p≥0.05. For exact statistical details and numerical data values in the scatter plot refer to S1 and S2 Data. (TIF) [file pgen.1009938.s004.tif]

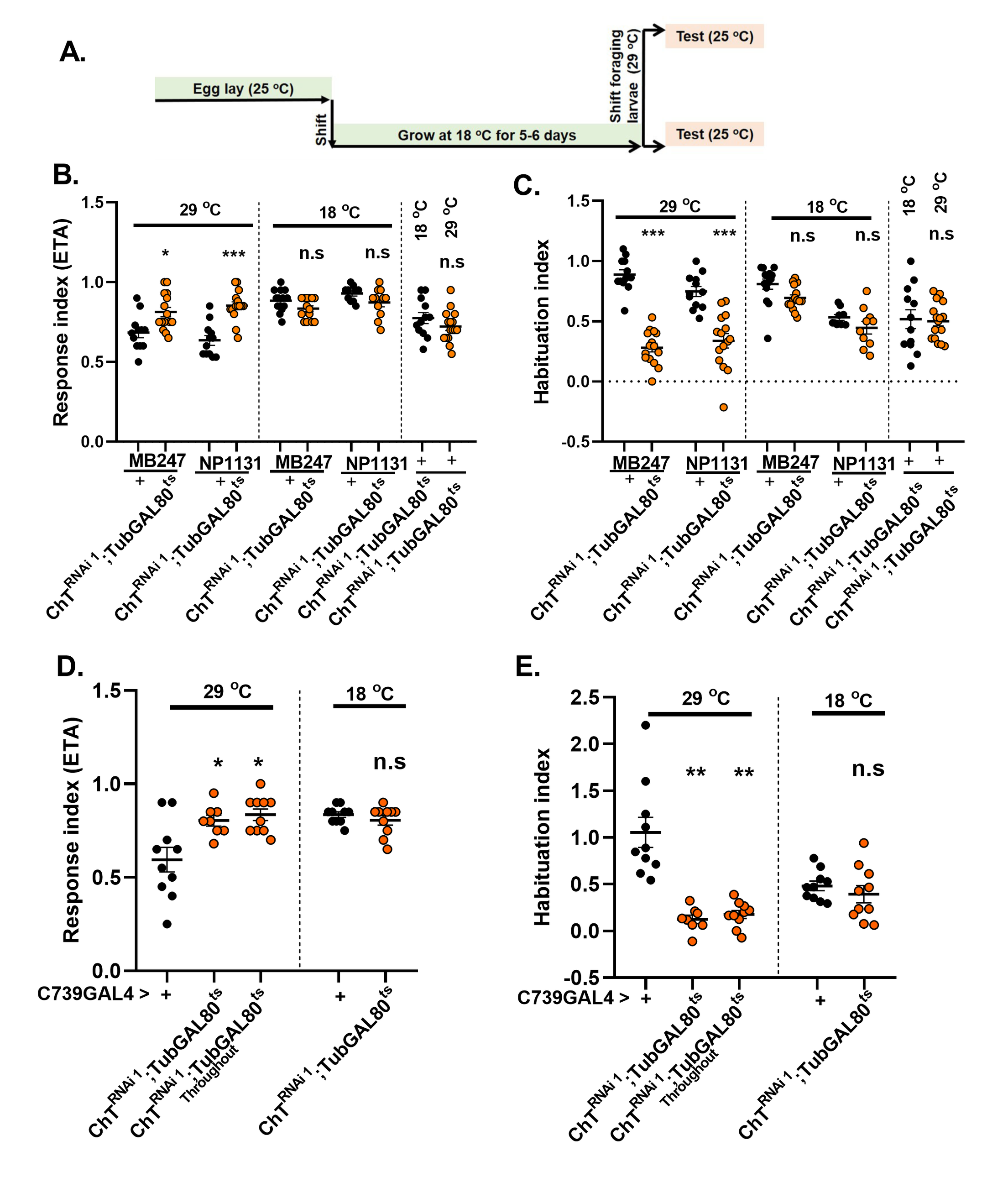

Supplement: S5 Fig — A) Schematic shows specific time window of temperature shift for TARGET system using TubGAL80ts. (B) response index, and (C) habituation index of group of larvae with genotype MB247GAL4>ChTRNAi1;TubGAL80ts (orange circles) compared to control MB247GAL4>+ (Black circles), NP1131GAL4>ChTRNAi1;TubGAL80ts (orange circles) compared to control NP1131GAL4>+ (Black circles) at 29°C and 18°C, ChTRNAi1;TubGAL80ts > + at indicated temperatures of 29°C and 18°C. (C) response index, and (D) habituation index in genotypes C739GAL4>ChTRNAi1;TubGAL80ts (orange circles) where ChT is specifically knocked down in 3rd instar developmental window or throughout development at 29°C compared to control C739GAL4>+. Scatter plot also shows response index and habituation index in genotypes C739GAL4>ChTRNAi1;TubGAL80ts (orange circles) compared to C739GAL4>+ at 18°C.Knockdown of ChT in α/β (marked by MB247 and C739) and γ lobe neurons (marked by NP1131) specifically in 3rd instar development window shows enhanced chemotaxis and reduced habituation. Each N in scatter plot represent one experiment performed with a group of 40 larvae. Each data group was analysed for normal distribution using Shapiro-Wilk test. Statistical significance was determined by two-tailed unpaired t-test (parametric) with Welchs correction. *** represent p≤0.0001, ** represent p≤0.001, n.s means statistical non-significance when p≥0.05. For exact statistical details and numerical data values in the scatter plot refer to S1 and S2 Data. (TIF) [file pgen.1009938.s005.tif]

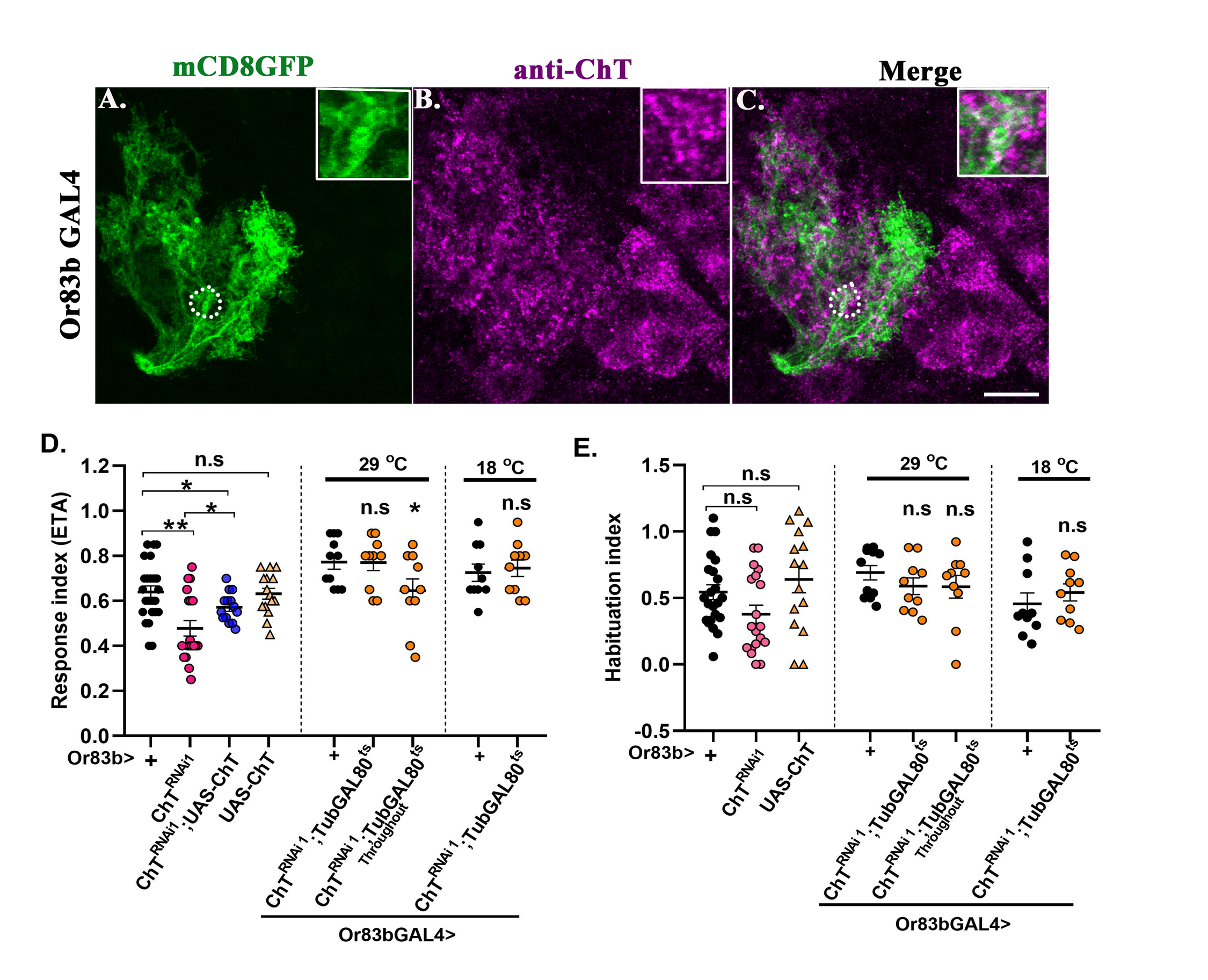

Supplement: S6 Fig — (A-C) Immunostained images of larval olfactory sensory neurons in 3rd instar dissected larval brain marked by expression of mCD8GFP using Or83bGAl4 driver in genotype Or83bGAL4>UAS-mCD8GFP (green), costained with anti-ChT (magenta), merge (colocalised regions of mCD8GFP and ChT appear as white). Inset shows a cropped and zoomed image of colocalised (mCD8GFP and ChT) terminal of OSNs, encircled by white dotted line. The image shown is a representative image of 3–5 brain lobes. Scale bar 50 μm (D) Response index, and (E) habituation index towards ETA of genotypes Or83bGAL4>+ (black circles), Or83bGAL4>ChTRNAi1 (pink circles), Or83bGAL4>ChTRNAi1;UAS-ChT (blue circles) and Or83bGAL4>UAS-ChT (yellow triangles). Or83bGAL4>ChTRNAi1;TubGAL80ts (orange circles) where ChT is specifically knocked down in 3rd instar developmental window or throughout development at 29°C compared to control C739GAL4>+. Scatter plot also shows response index and habituation index in genotypes Or83bGAL4>ChTRNAi1;TubGAL80ts (orange circles) compared to Or83bGAL4>+ at 18°C. Each N in scatter plot represent one experiment performed with a group of 40 larvae. Each data group was analysed for normal distribution using Shapiro-Wilk test. Statistical significance was determined by two-tailed unpaired t-test (parametric) with Welchs correction. ** represent p≤0.001, n.s means statistical non-significance when p≥0.05. For more statistical details and numerical data values in the scatter plot refer to S1 and S2 Data. (TIF) [file pgen.1009938.s006.tif]

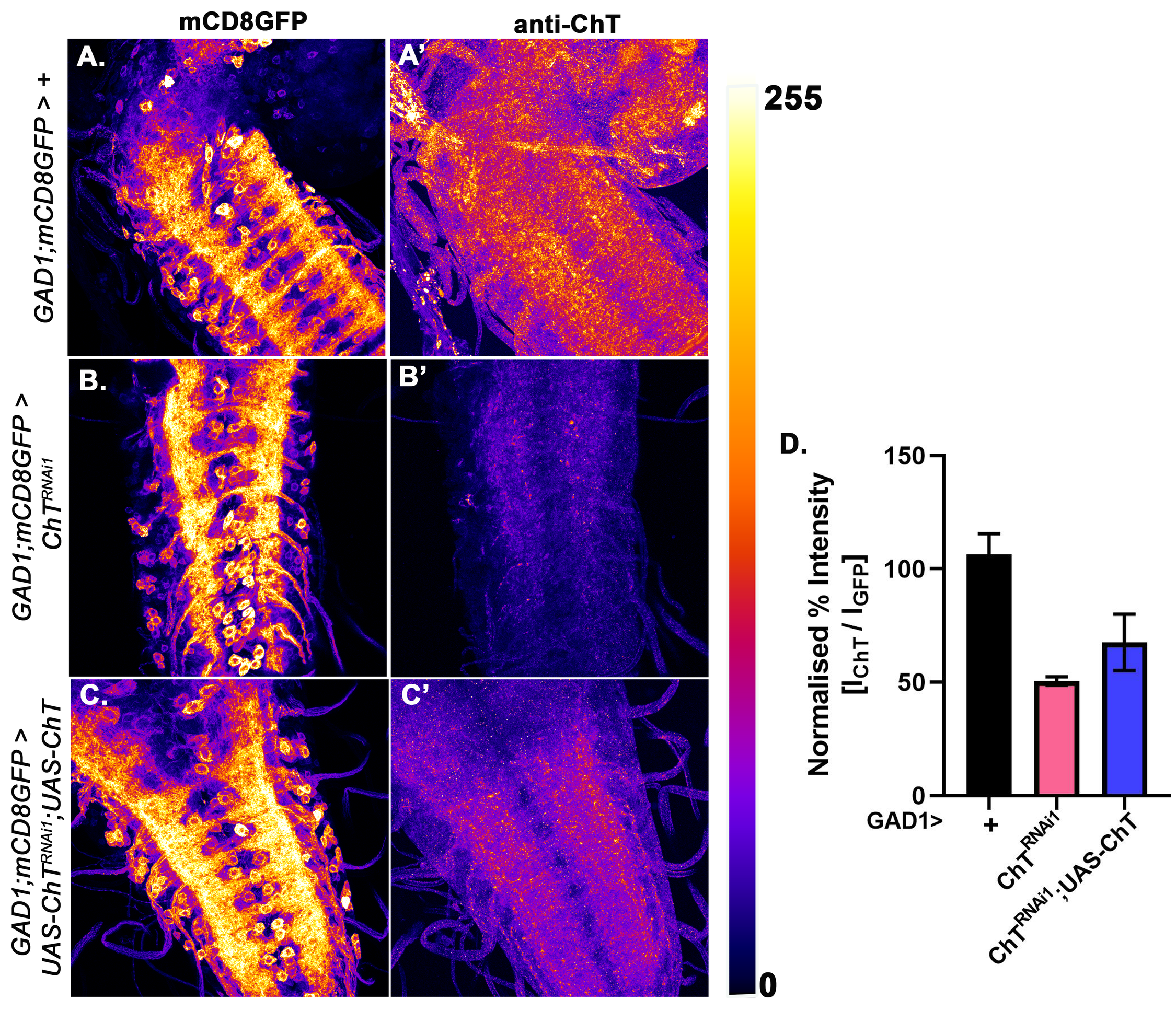

Supplement: S7 Fig — Intensity quantification of anti-ChT fluorescence signal normalised to mCD8GFP intensity signal driven by GAD1GAL4. One representative image from each genotype is shown as:(A-A’) GAD1;mCD8GFP>+, (B-B’) GAD1;mCD8GFP> UAS-ChTRNAi1, (C-C’) GAD1;mCD8GFP> UAS-ChTRNAi1; UAS-ChT. All samples were imaged at similar imaging parameters. The images are shown as fire LUT map showing the scale of colours from minimum 0 pixel intensity to maximum 255 pixel intensity. The scale of intensity colours is shown on the right. (D) Bar graph shows quantification of ChT intensity signals (6–10 ROI of 120x120 pixels per brain in regions marked by GAD1GAL4>mCD8GFP) normalised to GFP intensity signals in the corresponding regions. Each bar represents normalised % intensity value (IChT/IGFP) from indicated genotypes. For each genotype N = 3 brains were taken. For numerical data values representing the bar graph refer to S1 Data. (TIF) [file pgen.1009938.s007.tif]
